# Triplet Formation in a 9,10-Bis(phenylethynyl)anthracene Dimer and Trimer Occurs by Charge Recombination Rather than Singlet Fission

**DOI:** 10.1021/acs.jpclett.3c02050

**Published:** 2023-08-29

**Authors:** Rasmus Ringström, Zachary W. Schroeder, Letizia Mencaroni, Pavel Chabera, Rik R. Tykwinski, Bo Albinsson

**Affiliations:** † Department of Chemistry and Chemical Engineering, Chalmers University of Technology, Kemivägen 10, 412 96 Gothenburg, Sweden; ‡ Department of Chemistry, University of Alberta, Edmonton, Alberta, Canada T6G 2G2; § Department of Chemistry Biology and Biotechnology, University of Perugia, via elce di sotto n. 8, 06123 Perugia, Italy; ∥ The Division of Chemical Physics and NanoLund, Lund University, 22100 Lund, Sweden

## Abstract

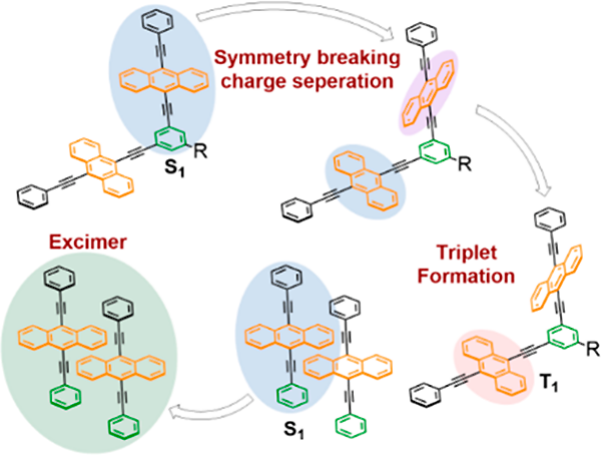

We present an experimental study investigating the solvent-dependent dynamics of a 9,10-bis(phenylethynyl)anthracene monomer, dimer, and trimer. Using transient absorption spectroscopy, we have discovered that triplet excited state formation in the dimer and trimer molecules in polar solvents is a consequence of charge recombination subsequent to symmetry-breaking charge separation rather than singlet fission. Total internal reflection emission measurements of the monomer demonstrate that excimer formation serves as the primary decay pathway at a high concentration. In the case of highly concentrated solutions of the trimer, we observe evidence of triplet formation without the prior formation of a charge-separated state. We postulate that this is attributed to the formation of small aggregates, suggesting that oligomers mimicking the larger chromophore counts in crystals could potentially facilitate singlet fission. Our experimental study sheds light on the intricate dynamics of the 9,10-bis(phenylethynyl)anthracene system, elucidating the role of solvent- and concentration-dependent factors for triplet formation and charge separation.

Singlet fission (SF) is a multiple exciton generation process in which a singlet excited state shares its excitation energy with a neighboring ground state molecule to produce two triplet excited states.^1^ SF has garnered significant attention in recent years due to its potential to enhance the efficiency of photovoltaic devices by utilizing the excess energy from high-energy photons that would otherwise be lost as heat.^2,3^ To integrate SF with conventional photovoltaics, certain essential requirements must be met. These requirements include photostability to ensure the longevity of the material under prolonged light exposure as well as energy alignment of the resulting triplet states with the bandgap of the photovoltaic device to enable efficient charge separation and collection. Meeting these criteria is crucial for harnessing the full potential of SF in practical applications. One molecule that exhibits promising characteristics to fulfill these requirements is 9,10-bis(phenylethynyl)anthracene (BPEA). BPEA is a robust and photostable industrial dye with a triplet energy of 1.2–1.3 eV,^4^ and it has demonstrated the ability to undergo SF both in thin films and in nanoparticles.^5−8^ However, studies using molecular dimers mimicking the slip-stacked nature of solid-state packing have suffered from low yields of SF due to competing excimer formation.^9^ SF in BPEA and other materials has been shown to be highly sensitive to the interchromophore orientation and distance.^8,10−12^ Because calculations suggest that the basic energetic requirement *E*(S_1_) ≥ 2*E*(T_1_)^4,13^ for SF is fulfilled for BPEA, it is possible that there are dimer designs that can suppress the excimer decay pathway and promote SF. Furthermore, if the calculations are underestimating the triplet energy and SF is slightly endothermic, it might be possible to enhance the rate of SF by increasing the number of chromophores as has been seen for other endothermic SF systems.^14,15^

Herein, we have synthesized and investigated a BPEA dimer (BPEAdim) and trimer (BPEAtri) connected in the meta position of the central phenylene unit. The molecular structures are shown in Scheme 1 together with the molecular structure of the monomer (BPEAmono). Using fluorescence spectroscopy measurements under total internal reflection (TIR) conditions on highly concentrated solutions of BPEAmono, we found that the main decay pathway of the monomer is by excimer formation. In contrast, BPEAdim and BPEAtri show no evidence of excimer formation, and the formation of the triplet excited state is observed. The triplet excited state formation is, however, not a consequence of SF, but rather by charge recombination from the symmetry breaking charge separated state (CSS) in polar solvents.

**Scheme 1:**
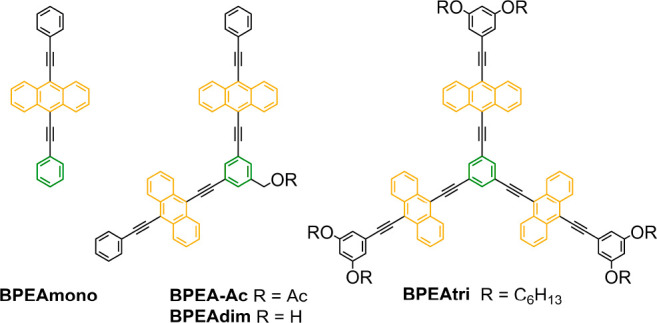
Molecular Structures of BPEAmono, BPEAdim, and BPEAtri

The syntheses of BPEAdim and BPEAtri share 9,10-dibromoanthracene as a common precursor (Scheme 1; see the Supporting Information for details). Substitution at the central benzene or peripheral phenyl moieties of BPEAdim and BPEAtri, respectively, was necessary to provide solubility and facilitate purification. The *m*-phenylene-linked BPEAdim was synthesized based on a two-step procedure from known building blocks. The precursor to BPEAdim, acetoxy-protected dimer BPEA-Ac (Scheme 1), was difficult to purify, and the crude reaction mixture was thus carried through the subsequent deacetylation to enhance the polarity of the dimer and facilitate purification. Gratifyingly, BPEAdim was obtained pure through a sequence of precipitation from the hydrolysis reaction mixture, column chromatography on silica gel, and recrystallization from CH_2_Cl_2_/MeOH. Compound BPEAtri was prepared using a 3-fold Sonogashira coupling reaction with the appropriate brominated anthracene and 1,3,5-triethynylbenzene building blocks. The product was purified via a sequence of size exclusion chromatography, silica gel column chromatography, and precipitation from a saturated solution of toluene via the addition of hexanes at −30 °C. Both BPEAdim and BPEAtri are stable solids under ambient conditions. Refer to Section S8 in the Supporting Information for further details.

Figure 1 displays the absorption and emission spectra of dilute solutions (∼5–10 μM) in tetrahydrofuran (THF). The absorption and emission spectra of BPEAmono, BPEAdim, and BPEAtri show only minor variations. The S_1_ energy of BPEAdim, as determined from the intersection of the absorption and emission signals, is red-shifted 0.027 eV relative to the monomer at 474 nm, and BPEAtri is shifted by an additional 0.027 eV. The unstructured absorption spectrum, in contrast to the structured emission, is attributed to excited state planarization, which has previously been studied in detail.^16^

**Figure 1:**
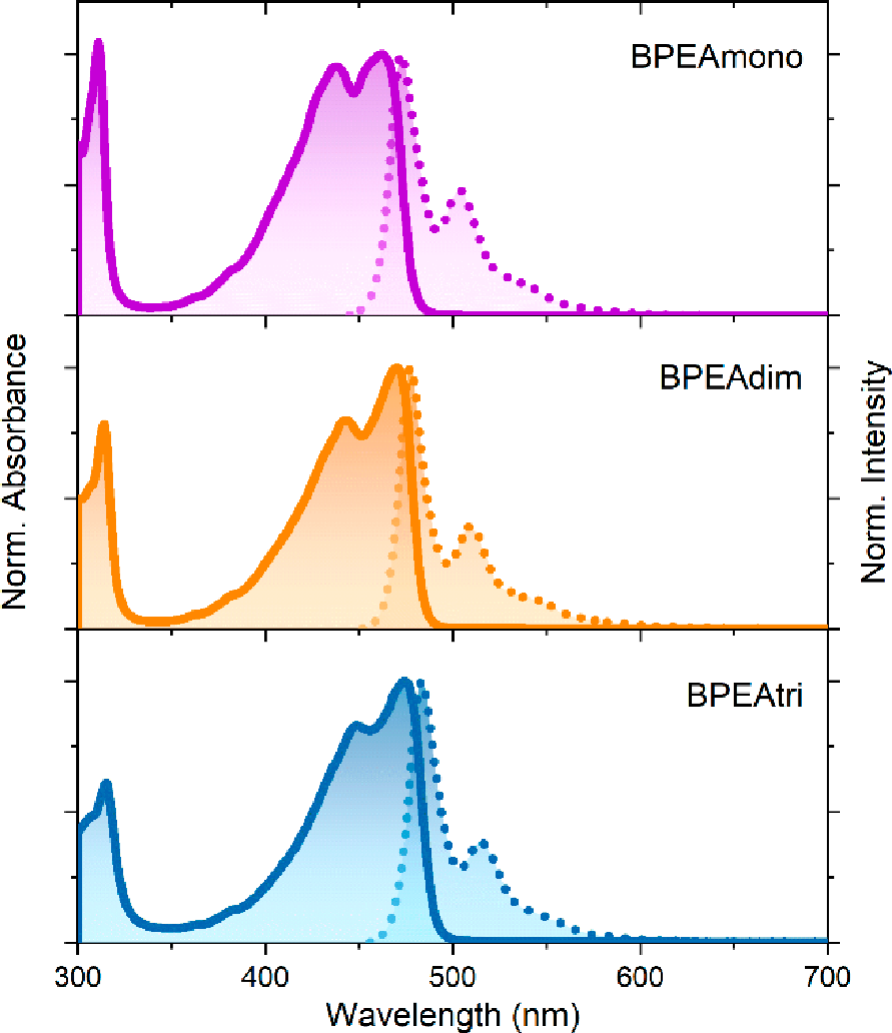
Steady state absorption (solid line) and emission (dotted line) of BPEAmono, BPEAdim, and BPEAtri in tetrahydrofuran (THF).

The photophysical properties of BPEAmono have been initially examined in both dilute and concentrated solutions to assess its suitability for SF. In dilute solution, BPEAmono has a fluorescence quantum yield close to unity and decays monoexponentially with a lifetime of approximately 3.4 ns (Figure 2a). Conversely, highly concentrated solutions show significantly quenched emission, as evidenced by the data presented in Figure 2a. To gain further insight into the behavior of BPEAmono in concentrated solutions, emission measurements were conducted under total internal reflection (TIR) conditions. These measurements, in conjunction with transient absorption analysis, indicate that excimer formation rather than SF serves as the primary decay pathway in the concentrated solutions. Section S2 in the Supporting Information provides a comprehensive account of the analysis, and Section S3 outlines the TIR technique employed. The formation of excimers is a plausible explanation for the poor upconversion quantum yield previously reported for BPEAmono.^4^

**Figure 2:**
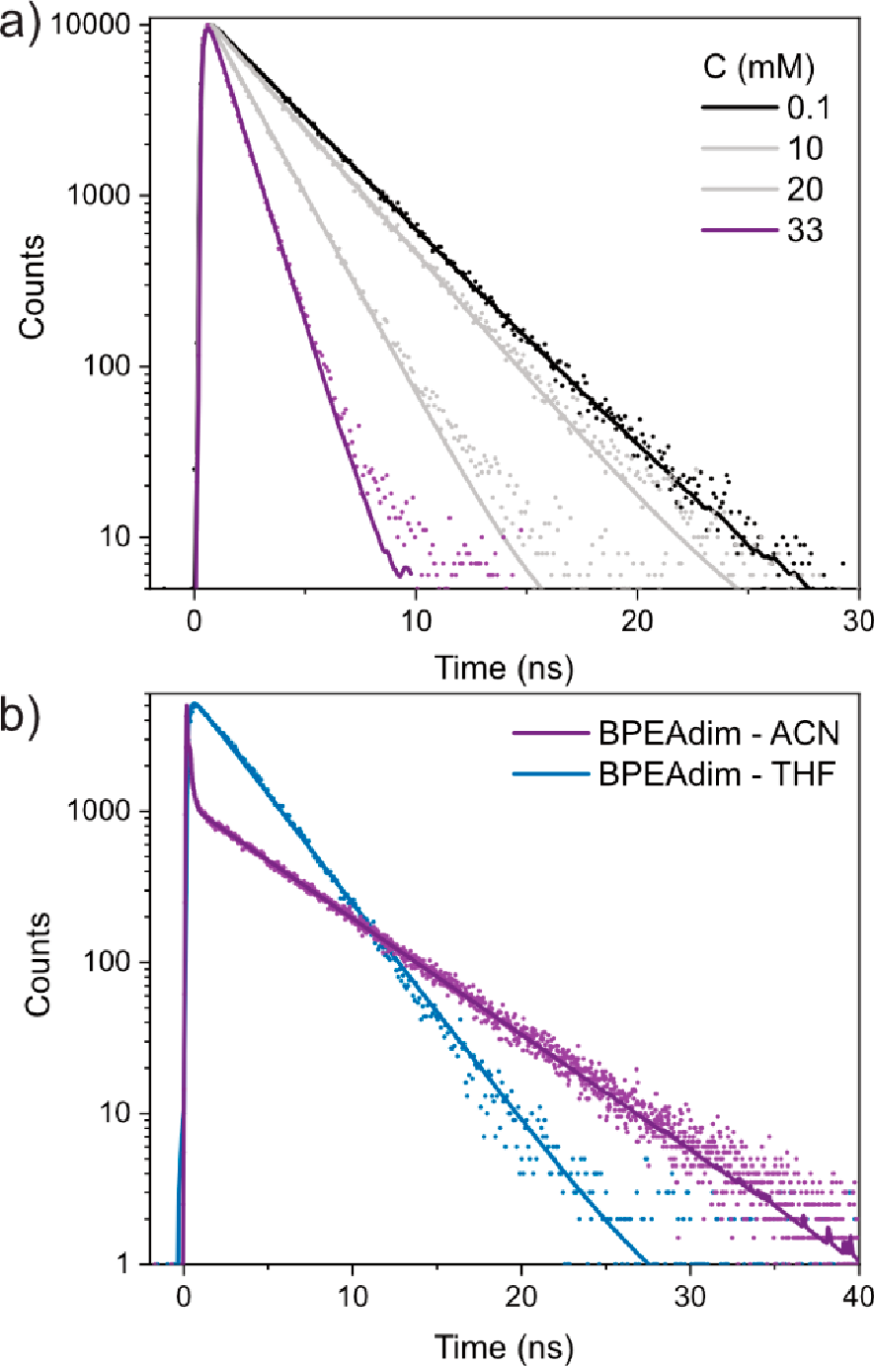
Time-resolved emission obtained with an excitation wavelength of 405 nm. (a) BPEAmono in THF for different concentrations (C) monitored at 473 nm. (b) BPEAdim in acetonitrile (ACN) and THF monitored at 480 nm. Fits for lifetimes are presented in Tables S1 and S3 for BPEAmono and BPEAdim, respectively.

The emission spectra of BPEAdim and BPEAtri closely resemble that of BPEAmono in a dilute solution (Figure 1). Notably, there is no evidence of excimer formation, as observed in other BPEA dimers^9^ as well as for BPEAmono at high concentration in the present study. Additionally, the emission lifetime of BPEAdim and BPEAtri in nonpolar solvents is similar to that of BPEAmono, indicating no substantial quenching of the singlet excited state. In polar solvents, however, emission of BPEAdim decays significantly within the response function of our time-correlated single photon counting (TCSPC) instrument (Figure 2b). To gain a better understanding of the quenching mechanism, we employed femtosecond transient absorption (fsTA). Figure 3 presents the fsTA spectra and the species associated spectra of BPEAdim in THF, showcasing a spectral evolution comparable to that of BPEAmono (Figure S4). Initially, the S_1_ absorption, centered at 560 nm, evolves within the first few picoseconds, corresponding to excited state planarization of the molecule. After planarization, the spectral features uniformly decay across the entire spectral range with a lifetime of 3.2 ns, corroborating well the results from TCSPC (Figure 2b and Table S3). The fsTA spectra of BPEAdim in THF have been analyzed using a two-component sequential decay model shown in the inset of Figure 3b. By employing singular value decomposition (SVD) and global analysis with the KiMoPack software,^17^ we can extract the spectral components and kinetics, as illustrated in Figures 3b and 3c. When BPEAdim is studied in the more polar solvent acetonitrile (ACN), we observe similar planarization as in THF, which is documented by the initial growth of the signal at 560 nm (Figure 4a). Following planarization, a new species emerges concomitantly with the decay of the singlet excited state, giving rise to broad excited state absorption at 640 nm. Based on the similarity with the spectra of the radical anion obtained through spectroelectrochemistry (Figure 4f and additional details in Section S4) and previous reports on BPEA dimers, we assign this new species as the CSS. Note that the spectroscopic signature of the CSS is a combination of the spectra of the radical anion and cation. For BPEAdim, however, the spectrum of the radical cation has very low molar absorptivity in the visible region compared to the anion (Figure S12). As the CSS state decays, a fourth state becomes discernible at 490 nm. This state exhibits a strong resemblance to the sensitized triplet excited state and has not been reported in previous studies on BPEA dimers.^18^ It is possible that this triplet has eluded detection due to the use of less polar solvents or, alternatively, to limitations in the length of delay stages, as the signal becomes apparent only at longer time delays. The fsTA spectra of BPEAdim in Figure 4a have been fit to the four-component model (Figure 4d). The spectral components and kinetics at selected wavelengths are displayed in Figures 4b and 4c, respectively. Previous studies on SF dimers with significant through-bond electronic coupling typically exhibit short triplet-pair lifetimes, ranging from several to hundreds of picoseconds, owing to the lack of triplet decorrelation and subsequent separation into free triplets.^19−23^ In contrast, the microsecond lifetime of the triplet excited state observed in our analysis (measured with nanosecond transient absorption, Figures S18 and S19), coupled with the emergence of the signal after the decay of the CSS, suggests the formation of one independent triplet excited state per molecule through charge recombination via CSS. Our findings thus support the hypothesis that the triplet formation is not a result of SF.^24^ Triplet formation within organic molecules via a CSS commonly ensues through one of two distinct mechanisms: spin–orbit charge transfer intersystem crossing (SOCT-ISC) and radical-pair intersystem crossing (RP-ISC).^25−27^ SOCT-ISC entails direct conversion of the initial ^1^CSS into the T_1_ state, involving a reverse electron transfer and a spin inversion process. This phenomenon is notably amplified when the constituent subunits assume a nearly perpendicular alignment. Such an orientation facilitates compensation for changes in electron spin angular momentum during ISC through adjustments in molecular orbital angular momentum. This intricate alignment is particularly evident in closely spaced dyads, where steric hindrance between subunits enforces an orthogonal arrangement, as observed in dyads linked by a single C–C bond.^28,29^ In contrast, the RP-ISC mechanism involves the emergence of an intermediate triplet charge-transfer state (^3^CSS) prior to charge recombination, leading to either the triplet state or the ground state. This mechanism is more prevalent in electron donor–acceptor dyads characterized by weak electronic coupling between their donor and acceptor subunits due to considerable spatial separation (e.g., >10–15 Å). This separation results in a minimal energy difference between ^1^CSS and ^3^CSS. Notably, certain studies posit the coexistence of both mechanisms.^30^ Distinguishing between SOCT-ISC and RP-ISC through fsTA spectroscopy proves challenging because radical pairs are central to both processes, yielding closely resembling optical spectral signatures. Consequently, we can only engage in qualitative speculation regarding the mechanism underlying our observations. The rate of triplet excited state formation seems to align with previous RP-ISC studies, as spin–orbit coupled mechanisms generally exhibit larger rates.^31^ Additionally, the interchromophore distance within BPEAdim exceeds 10 Å, further reinforcing the alignment with the RP-ISC mechanism. The relatively slow charge recombination rate toward the ground state, in comparison to triplet formation, may be explicable through the substantial negative Gibbs free energy shift accompanying ground state recombination. This scenario potentially gives rise to behavior consistent with the Marcus inverted region characteristics.

**Figure 3:**
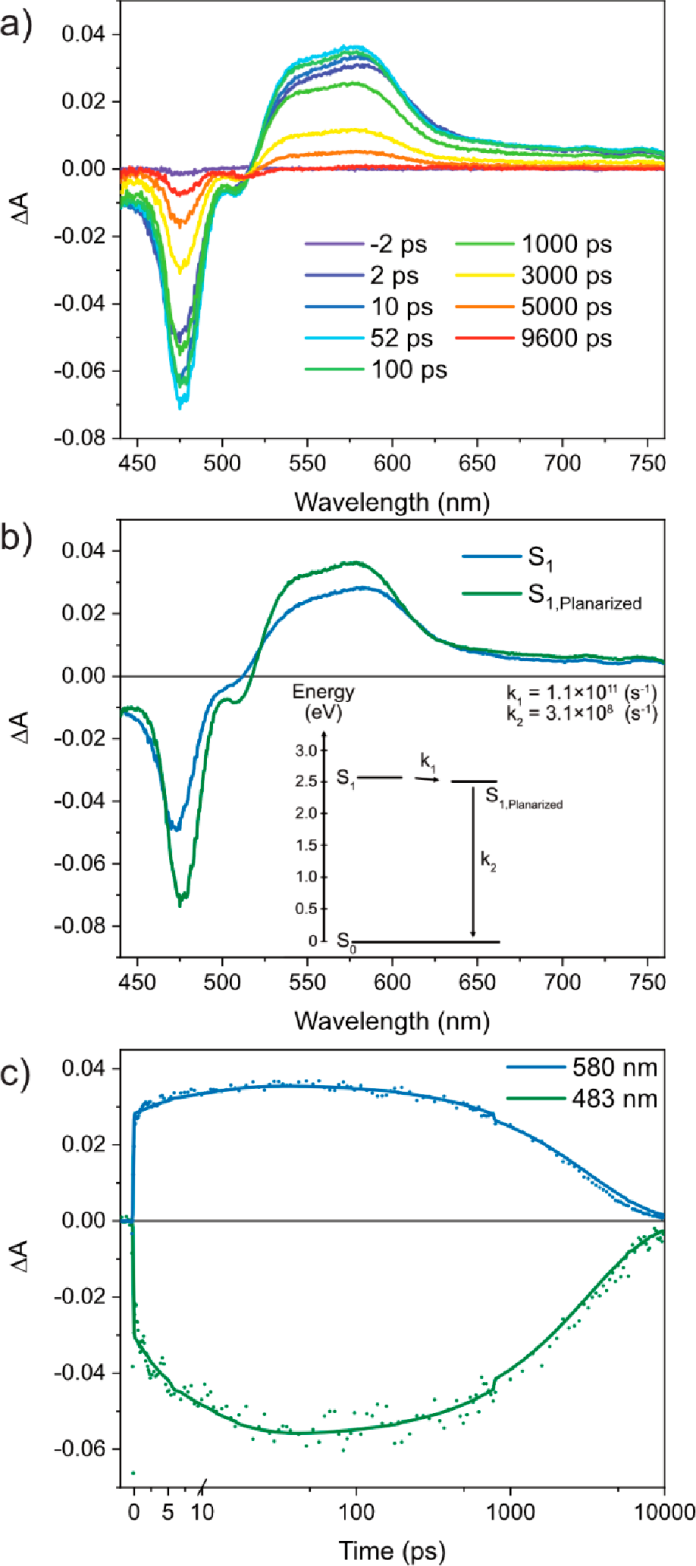
(a) fsTA analyses of BPEAdim in a dilute solution of THF (∼10 μM); excitation at 420 nm. (b) Species associated spectra of the singular value decomposition (SVD) analysis using the kinetic model shown in the inset. (c) Selected kinetics of ground-state bleach and stimulated emission at 483 nm and S_1_ excited state absorption at 580 nm with the model data shown as a solid line.

**Figure 4:**
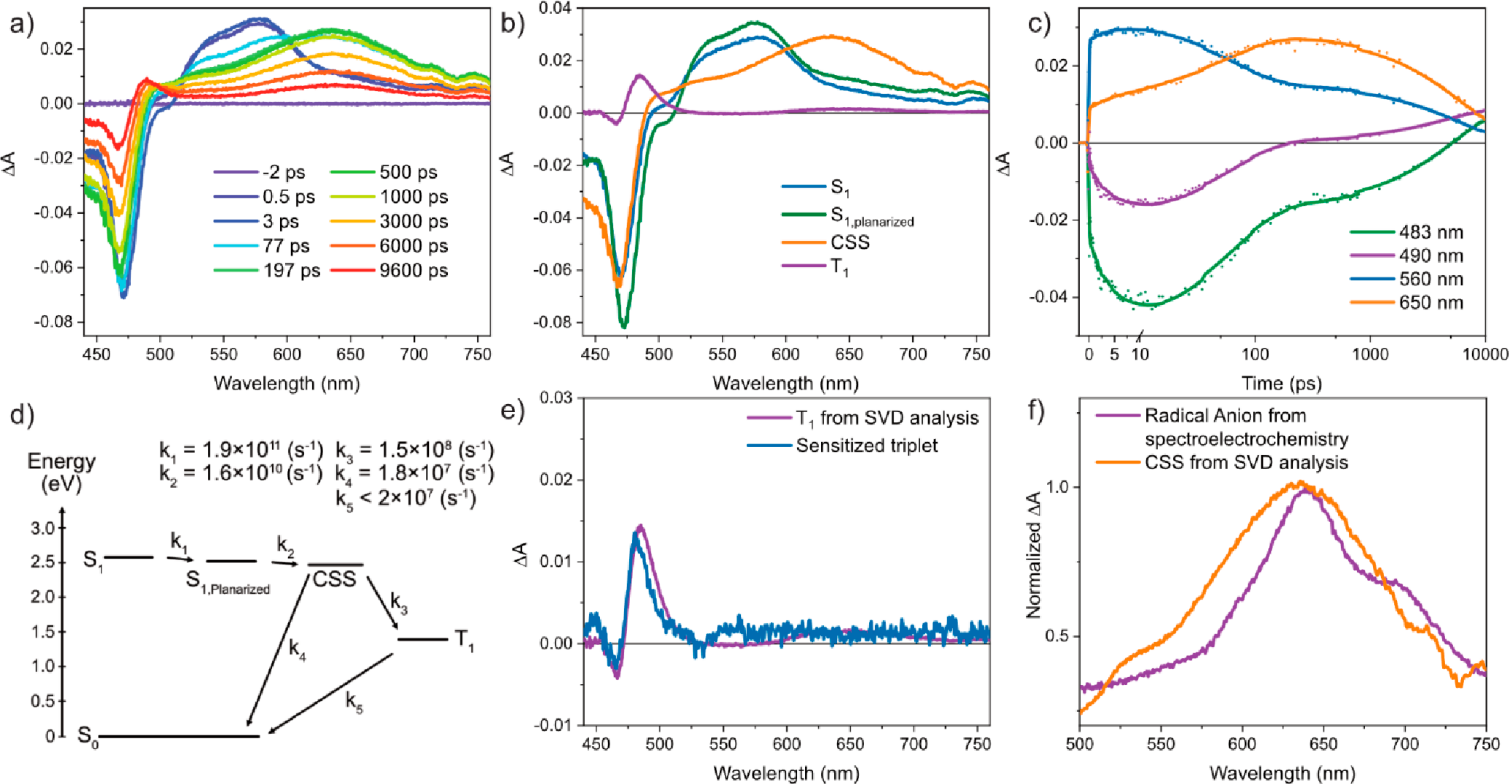
(a) fsTA analyses of a dilute solution (∼5 μM) of BPEAdim in ACN; excitation at 420 nm. (b) Species associated spectra of the SVD analysis using the model shown in panel d. (c) Selected kinetics of ground state bleach/T_1_ at 483 and 490 nm, S_1_ at 560 nm, and the CSS at 650 nm with the model data shown as a solid line. (d) Kinetic model and associated rate constants. The energy of the CSS was estimated based on the redox potentials of BPEAmono (see Section S4 for more details). (e) Comparison of the final spectral component from the singular value decomposition (SVD) analysis and kinetic model compared to the sensitized triplet absorption spectrum (see Section S7 for more details). (f) Comparison of the CSS spectral component with the radical anion obtained via spectroelectrochemical analysis (see Section S4 for more details). Note that the CSS spectrum is a combination of the radical anion and cation spectra, while for BPEAdim the spectrum of the radical cation has very low and broad molar absorptivity in the visible region compared with the anion (Figure S12).

It is noteworthy that the CSS observed in our study has a spectroscopic signature that is similar to what was previously attributed to a shifted and broadened S_1_ absorption in BPEA thin films.^5^ Based on this observation, it is possible that the triplet formation observed for BPEA thin films could originate from a similar charge recombination process as we have proposed in the present study and not from SF, as previously proposed.^5^ The mechanism of charge recombination to form the triplet excited state has been proposed in other thin film systems, such as in the indigo derivative cibalackrot, in which case transient intermediates with charge-transfer character are also observed prior to triplet formation.^32^ It is also worth noting that the interpretation of the spectral feature as a shifted and broadened S_1_ absorption in BPEA thin films could be entirely accurate, given that the excited state absorption of various compounds, including polyacenes, are known to differ significantly between solution and film states.^33−37^

The photophysical behavior of BPEAtri appears to be very similar to that of BPEAdim, and SF is not detected in dilute solution, regardless of solvent polarity. (Further details are provided in Section S6. BPEAtri was not investigated in ACN due to limited solubility.) Consequently, it seems that an additional BPEA moiety is insufficient to drive the seemingly slightly endothermic SF of BPEA. It is possible that a larger oligomer or a linear arrangement of chromophores could enhance the entropic driving force(s), as observed in other endothermic SF systems.^14,15^ In fact, fsTA measurements in highly concentrated solutions (25 mM) of BPEAtri in THF reveal a broad excited state absorption feature at 500 nm during later time delays that matches the sensitized triplet spectra (Figure S27). Although the absorption spectrum shows no evidence of significant scattering from aggregates, we posit that smaller aggregates may form, and these aggregates could facilitate SF. However, there is also evidence of excimer formation for BPEAtri as the emission lifetime depends on the probing wavelength, indicating the presence of at least two emissive species, similar to that observed for BPEAmono (Figure S23 and Table S8).

In this study, we investigated the photophysical properties of a BPEA monomer, dimer, and trimer. Our findings show that in highly concentrated solutions, BPEAmono exhibits excimer formation, and SF is not observed. The photophysics of BPEAdim and BPEAtri are primarily governed by symmetry-breaking charge separation, which outcompetes SF. Triplet excited states are, however, generated by charge recombination, where the CSS acts as an intermediate. Interestingly, there are indications of SF in highly concentrated solutions of the trimer as triplet excited state absorption is discernible without prior formation of the charge-separated state. This suggests that increasing the number of chromophores with larger oligomers, mimicking the larger chromophore count in crystals, may enhance the rate of SF for BPEA and potentially for other SF systems in solution.

## Supporting Information Available

Additional absorption, emission, transient absorption, spectroelectrochemical data, synthetic details, and full analysis of the compounds (PDF)
