# Supplementary material for: Triplet Formation in a 9,10-Bis(phenylethynyl)anthracene Dimer and Trimer Occurs by Charge Recombination Rather than Singlet Fission
Source: J Phys Chem Lett. 2023 Aug 29;14(35):7897–902. doi: 10.1021/acs.jpclett.3c02050 (PMC10494225; doi:10.1021/acs.jpclett.3c02050)
Supplement: Supplementary file 1 — jz3c02050_si_001.pdf [file jz3c02050_si_001.pdf]

Supporting information for

Triplet Formation in a 9,10-Bis(phenylethynyl)anthracene Dimer and Trimer Occurs by Charge Recombination Rather Than Singlet Fission

Rasmus Ringström[a], Zachary W. Schroeder[b], Letizia Mencaroni[c], Pavel Chabera[d], Rik R. Tykwinski[b], Bo Albinsson[a,*]

[a] Department of Chemistry and Chemical Engineering, Chalmers University of Technology, Kemivägen 10, 412 96 Gothenburg, Sweden.

[b] Department of Chemistry, University of Alberta, Edmonton, Canada T6G 2G2.

[c] Department of Chemistry Biology and Biotechnology, University of Perugia, via elce di sotto n. 8, 06123, Perugia, Italy.

[d] The Division of Chemical Physics and NanoLund, Lund University, 22100 Lund, Sweden.

Email: balb@chalmers.se

Table of Contents

1 Materials and Methods 1

2 Photophysical characterization of BPEAmono 3

3 Total internal reflection emission measurements 7

4 Electrochemistry 9

5 Photophysical characterization of BPEAdim 13

5.1 Steady-state absorption and emission 13

5.2 Time-resolved emission 14

5.3 fsTA 15

6 Photophysical characterization of BPEAtri 17

6.1 Steady-state absorption and emission 17

6.2 Time-resolved emission 18

6.3 fsTA 19

6.4 fsTA at high concentration 22

7 Triplet sensitization 23

8 Synthetic details 24

8.1 Experimental procedures 26

8.2 Experimental data and compound characterization 30

9 References 53

1 Materials and Methods

Optical spectroscopy: Steady-state absorption measurements were recorded with Varian-Cary 50 Bio and the emission spectra were obtained with a Spex Fluorolog 3 spectrofluorometer from JY Horiba. Please refer to

section 3 for experimental details of the total internal reflection measurements. Femtosecond transient absorption (fsTA) were performed with a Ti:sapphire oscillator (Mai-Tai, Spectra Physics) which was used as seed to a regenerative amplifier (Solstice Ace, Spectra Physics) pumped by a frequency-doubled diode-pumped Nd:YLF laser (Ascend, Spectra Physics). This produced pulses of around 60 fs duration (fwhm) at a 1 kHz repetition rate; the 800 nm output from the amplifier was split, and the two beams were used as pump and probe light. An optical parametric amplifier (TOPAS PRIME, Light Conversion Ltd.) was used to tune the pump wavelength to yield the appropriate excitation wavelengths (1μJ/pulse at the sample). The probe light was focused on a translating CaF_2 plate to generate a supercontinuum, and the pump beam was delayed with respect to the probe beam with an optical delay stage (range 0−10 ns). The supercontinuum was split into a probe and reference beam, and the probe beam overlapped with the pump at the sample. Residual fundamental was dumped using an 800 nm notch filter. The transmitted probe and reference beam were directed to optical fibers and detected by a CCD camera (iXon-Andor) operating synchronously with the 1 kHz laser. The transient spectra were obtained from the difference of the probe light divided by the reference with and without excitation of the sample by the pump beam; 2000 spectra were averaged per delay time using a custom LabVIEW program controlling the setup. Nanosecond transient absorption (nsTA) spectra and kinetic traces were recorded using an Edinburgh Instrument LP 980 spectrometer with a CCD camera (CCD, Andor DH320T-25F-03) or photomultiplier tube (PMT), respectively. The excitation source was a Spectra-Physics Nd:YAG laser (pulse width ~7 ns) coupled to a Spectra-Physics primoscan optical parametric oscillator (OPO).

Electrochemistry: The cyclic voltammetry measurements were performed with a CHI-potentiostat controlled using CHI650A software (version 11.15). Platinum electrodes were used as the working and counter electrodes and Ag/AgCl in saturated KCl was used as the reference electrode. The measurements were performed in thoroughly degassed (using Argon) dichloromethane (DCM) with 0.1 M tetra-n-butylammonium hexafluorophosphate $TBAPF_6$ (Sigma Aldrich) at a scan rate of 0.1 V/s. Ferrocene/Ferrocenium (Fc/Fc^+) was used as an external standard with $E_{1/2}$ at approximately 0.53 V vs. Ag/AgCl in DCM.

The spectroelectrochemical measurements were performed under the same conditions as the cyclic voltammetry measurements with the exception that a honeycomb electrode with a Pt working- and counter electrode was used. An Avantes AVALIGHT-DHC was used as the light source and was directed at the honeycomb electrode using fibreoptic cables. The spectra were recorded with an Avantes (AvaSpec-2048) fiberoptic spectrometer.

2 Photophysical characterization of BPEAmono

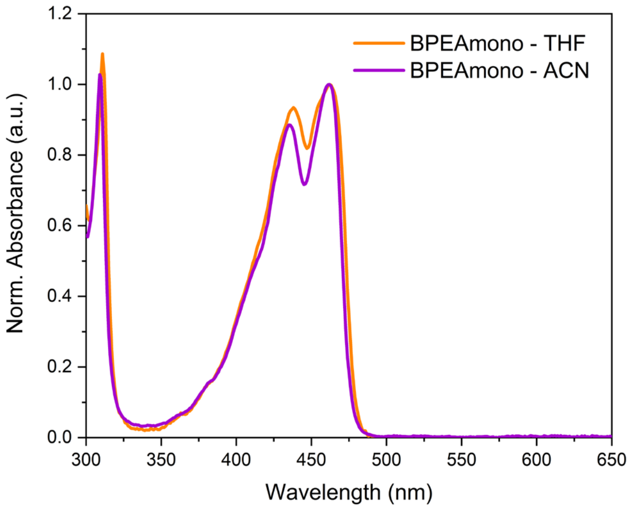

Figure S1. Normalized steady-state absorption of BPEAmono in THF and ACN.

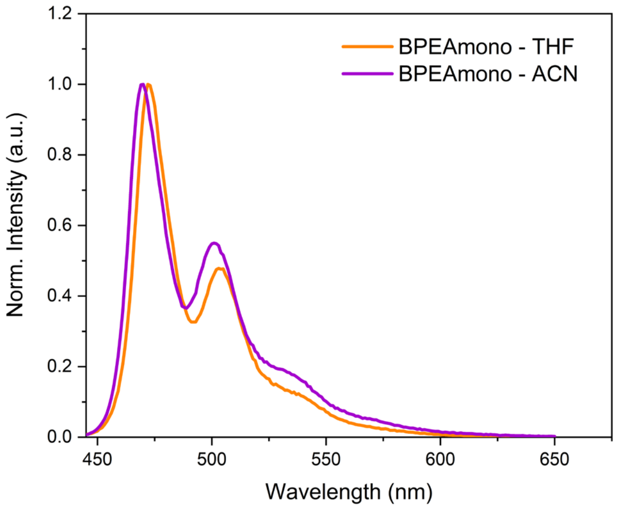

Figure S2. Normalized steady state emission spectra of BPEAmono in THF and ACN (excitation at 405 nm).

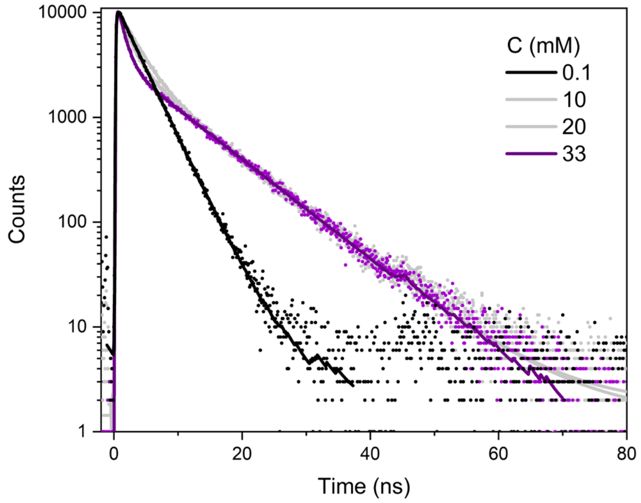

Figure S3. Time-resolved emission of BPEAmono in THF obtained with an excitation wavelength of 405 nm and monitored at 640 nm probing mainly the excimer emission lifetime, but also the tail of the prompt fluorescence. Refer to Table S1 for fitted lifetimes and relative amplitudes of the preexponential factors.

Table S1. Emission lifetime of BPEAmono in THF probed at 473 nm and 640 nm and relative amplitudes of the preexponential factor in%.

BPEAmono	473 nm	640 nm
C (mM)	τ (ns)	τ (ns)
0.1	3.3	3.4
10	3.0	3.0 (75%)
		9.5 (25%)
20	1.9	1.9 (71%)
		9.2 (29%)
33	1.1	1.1 (74%)
		9.0 (26%)

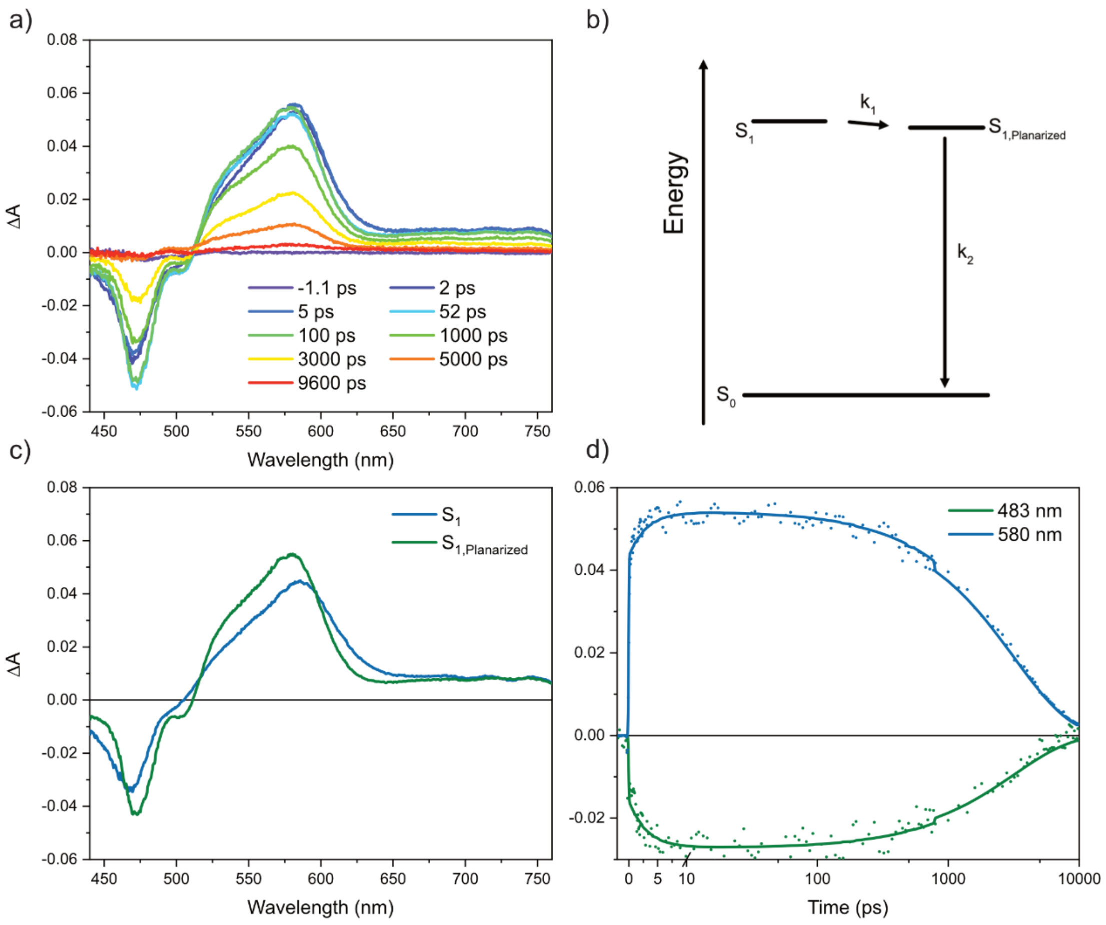

Figure S4. a) fsTA of a dilute solution of BPEAmono in THF with excitation at 420 nm. b) Schematic representation of the kinetic model. c) Species associated spectra of the SVD analysis using the model shown in b. d) Selected kinetics of ground-state bleach at 483 nm and S_1 at 580 with the model data shown as a solid line.

Table S2. Rate constants for BPEAmono in THF using the kinetic model in Figure S4b.

$1/k_1$ (ps)	$1/k_2$ (ps)
3.2	3210

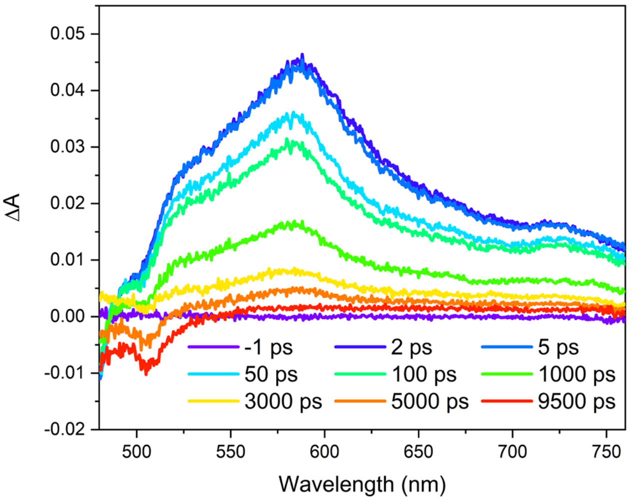

Figure S5. fsTA of a concentrated solution (30 mM) of BPEAmono in THF with excitation at 420 nm. Excimer emission can be observed at later time delays at around 525 nm.

3 Total internal reflection emission measurements

Total internal reflection (TIR) conditions at the interface between two mediums with refractive indices n_1 and n_2 can be realized if *i)* the first medium has a larger refractive index than the second and *ii)* the angle of the incident light (θ_i) of wavelength λ is larger than the critical angle (θ_c) as derived from Snell's law: $sin(\theta_c) = n_1/n_2$. Under these conditions, the intensity (*I*) of light that passes through the interface is called the evanescent wave. The evanescent wave decays exponentially according to $I(z) = I_0 e^{(-z/d)}$ where *z* is the distance and *d* is the penetration depth defined according to equation 1.[1]

$$d = \frac{\lambda}{4\pi\sqrt{n_1^2 \sin^2\theta_i - n_2^2}} \quad (1)$$

Our experimental setup consisted of a high refractive index half-sphere (n_1 = 2.0) placed in direct contact with the sample dissolved in THF (n_2 = 1.4072). The angle of incident light was 59° for all measurements which is well above the critical angle of 45°. An OBIS laser (λ_{ex} = 405 nm, Coherent, CW 2 mW) was used as the excitation source. The TIR emission was collected through an optical fiber and the emission spectra was collected with an Avantes (AvaSpec-2048) fiberoptic spectrometer. The sample holder used was a 96 µL 3D-printed concave cylinder made of nylon fibers to be resistant to THF. Under these conditions the penetration depth was calculated to be 32.9 nm. Consequently, the intensity has decreased to 1/e of the original value at 32.9 nm. After only 100 nm the intensity has dropped by more than 95% which ensures that the second order inner-filter effects are minimized.

Figure S6 demonstrates the difference in second order inner filter effects of a measurements conducted with and without the high refractive index (HRI) crystal at 15 mM. Figure S7 shows a series of emission measurements using the TIR emission setup on different concentrations of BPEAmono. At higher concentrations the excimer emission becomes more prominent.

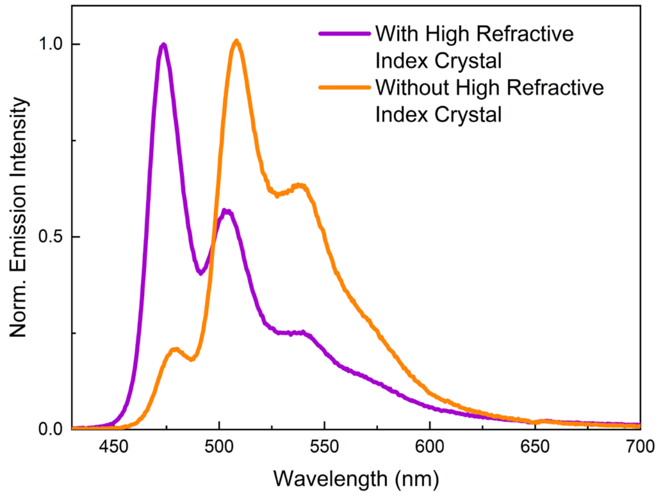

Figure S6. The normalized emission of 15 mM BPEAmono dissolved in THF with and without the high refractive index (HRI) crystal (excitation at 405 nm).

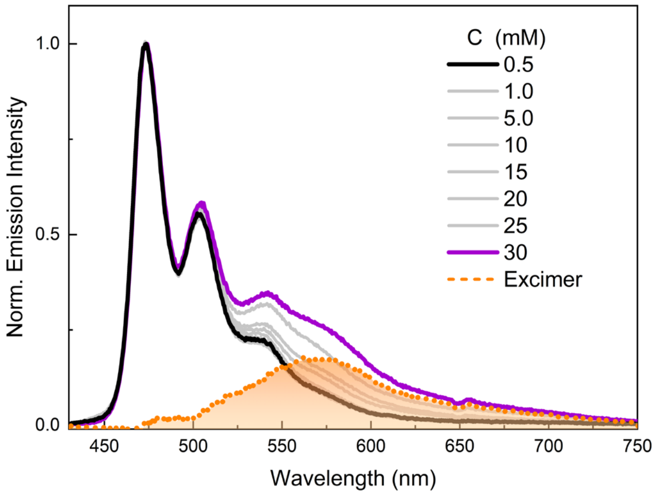

Figure S7. Concentration effect on the emission spectra of BPEAmono in THF detected by the TIR technique with excitation at 405 nm. The excimer emission (orange profile) was obtained by subtracting the emission profile of the 0.5 mM sample from the 30 mM sample.

4 Electrochemistry

BPEAmono displays a quasi-reversible reduction at $E_{1/2}$ = –1.85 V vs. Fc/Fc^+ and the first oxidation wave, which is irreversible, and occurs at +0.75 V vs. Fc/Fc^+. The small signal at ~ –0.2 V has been observed in previous reports[2] and is likely related to the degradation product of the irreversible oxidation wave. The oxidation and reduction potential of BPEAdim are only slighted shifted relative to BPEAmono as observed in Figure S10 and Figure S11. It was not possible to measure a well-resolved cyclic voltammogram of BPEAtri. However, the marginal shift of BPEAdim compared to BPEAmono together with the similarity of the radical anion and cation spectra of BPEAtri with BPEAmono and BPEAdim indicates that the oxidation and reduction occur at roughly the same potential for all three compounds.

The energy of the charge separated state of BPEA was estimated using equation 2[3,4]:

$$E(CSS) = E_{ox}(D) - E_{red}(A) - \frac{e^2}{4\pi\varepsilon_0\varepsilon_s R_{DA}} + \left(\frac{e^2}{2}\right)\left(\frac{1}{r_D} + \frac{1}{r_A}\right)\left(\frac{1}{4\pi\varepsilon_0\varepsilon_s} - \frac{1}{4\pi\varepsilon_0\varepsilon_s^p}\right) \qquad (2)$$

Here, $E_{ox}(D)$ and $E_{red}(A)$ are the oxidation and reduction potentials of the donor and acceptor, respectively. e is the elementary charge, $\varepsilon_0(= 8.854 \cdot 10^{-12}\ As/Vm)$ is the permittivity of vacuum, ε_s is the static dielectric constant of the solvent the electrochemical measurements were performed in (CH_2Cl_2) and ε_s^p is the dielectric constant of the solvent the photophysical measurements were measured. The center-to-center donor acceptor distance R_{DA} was estimated as the center of the first anthracene-moiety to the center of the other (14 Å). $r_D$and r_A was estimated as the center of the anthracene to the hydrogen atom at the end of the phenyl-unit (8 Å). Based on this, the energy of the CSS could be estimated to 2.42 eV in ACN. The energy of the CSS without the final term (which accounts for the difference in dielectric constant of the solvents used) is almost isoenergetic with the S_1 state. The dielectric constant of CH_2Cl_2, which the CV-measurements were conducted in is 8.93, which is slightly higher than that of THF (7.58). It is therefore reasonable to assume that the CSS energy is slightly higher than S_1 in THF. This also corroborates well with the experimental observations.

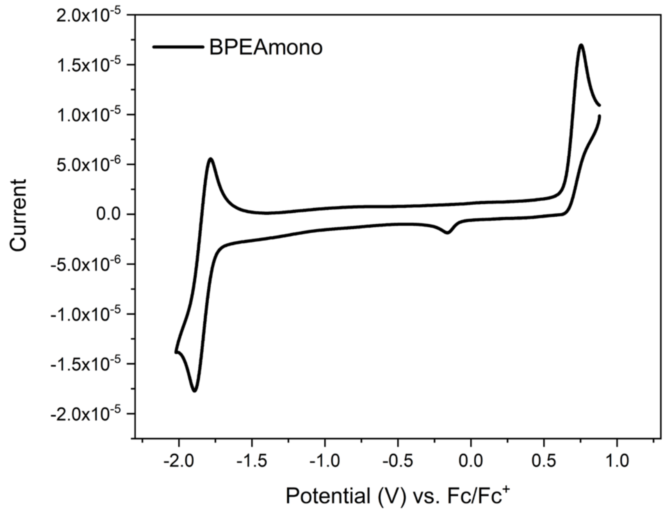

Figure S8. Cyclic voltammogram of BPEAmono in CH_2Cl_2 reported vs Fc/Fc^+.

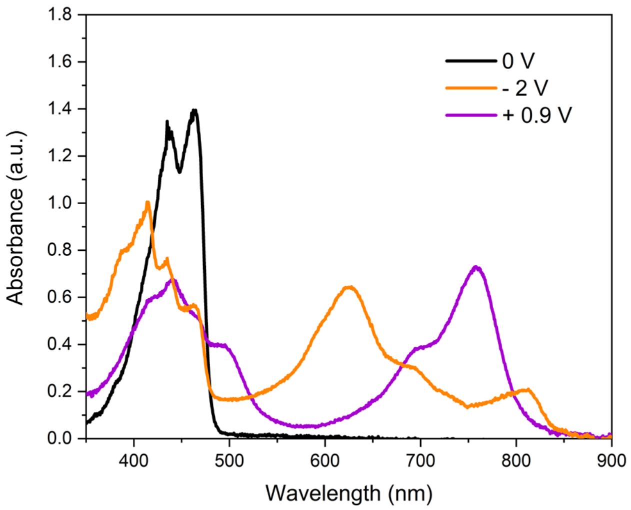

Figure S9. Absorption spectra of neutral BPEAmono (black), radical anion (orange) obtained by applying a potential of –2 V (vs Fc/Fc⁺), and the radical cation (purple) obtained by applying a potential of +0.9 V (vs Fc/Fc⁺) in CH_2Cl_2.

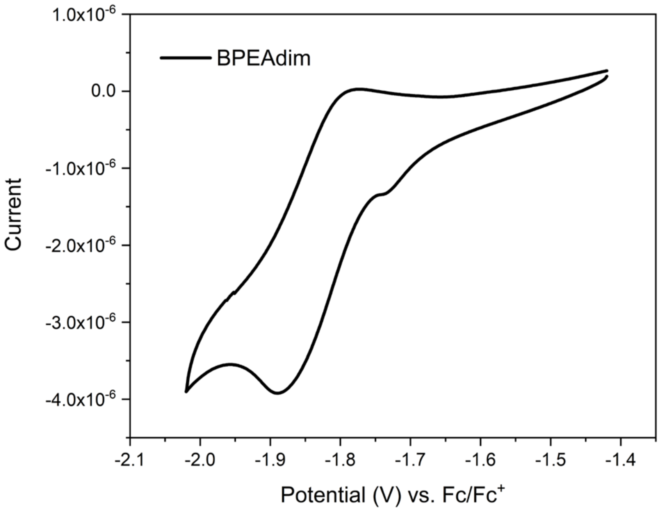

Figure S10. Reductive cyclic voltammogram of BPEAdim in CH_2Cl_2 reported vs Fc/Fc^+.

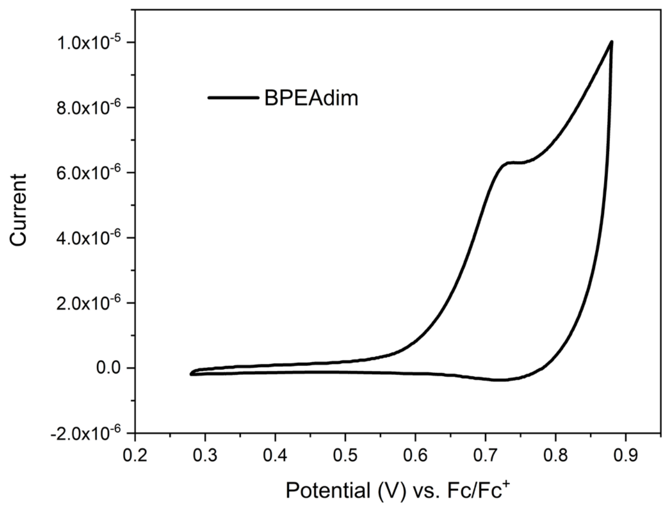

Figure S11. Oxidative cyclic voltammogram of BPEAdim in CH_2Cl_2 reported vs Fc/Fc^+.

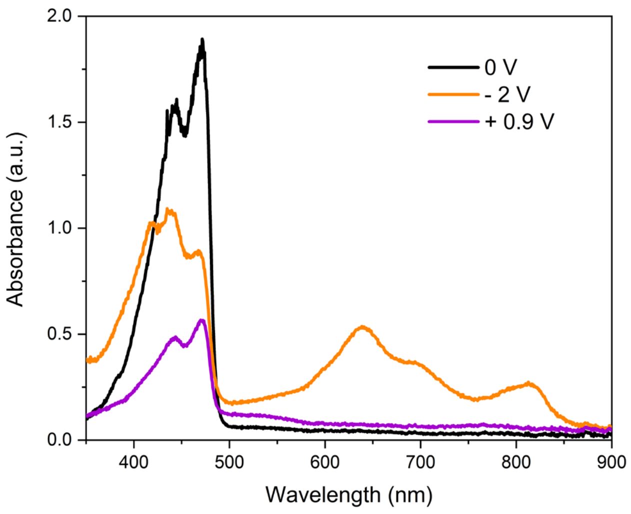

Figure S12. Absorption spectra of neutral BPEAdim (black), radical anion (orange) obtained by applying a potential of –2 V (vs Fcs/Fc^+), and the radical cation (purple) obtained by applying a potential of +0.9 V (vs Fc/Fc^+) in CH_2Cl_2.

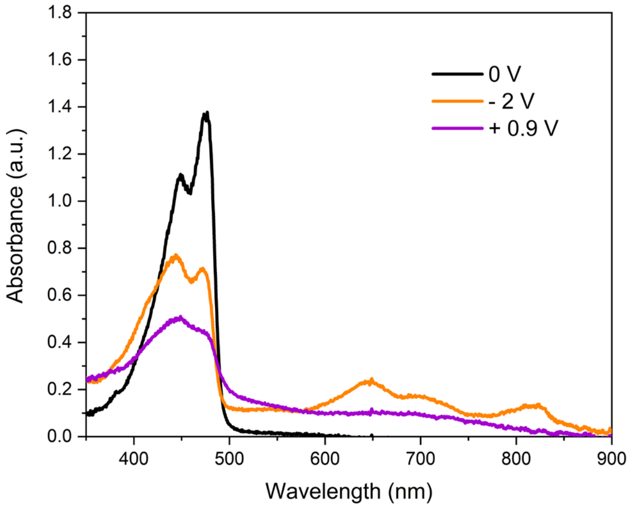

Figure S13. Absorption spectra of neutral BPEAtri (black), radical anion (orange) obtained by applying a potential of –2 V (vs Fc/Fc^{+}), and the radical cation (purple) obtained by applying a potential of +0.9 V (vs Fc/Fc^{+}) CH_2Cl_2.

5 Photophysical characterization of BPEAdim

5.1 Steady-state absorption and emission

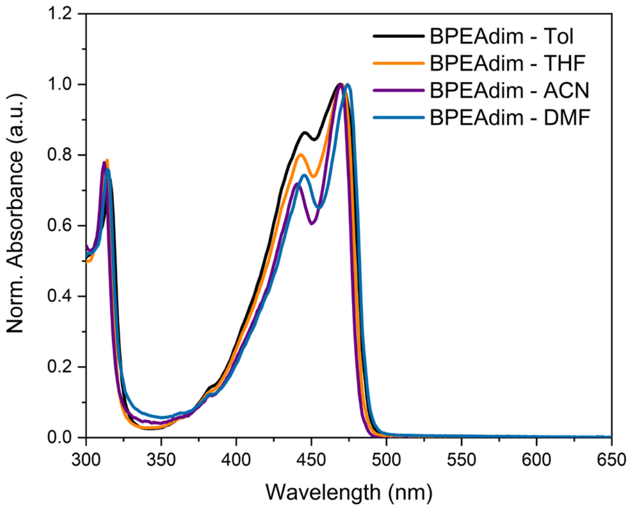

Figure S14. Normalized steady-state absorption of BPEAdim in toluene, THF, ACN, and DMF.

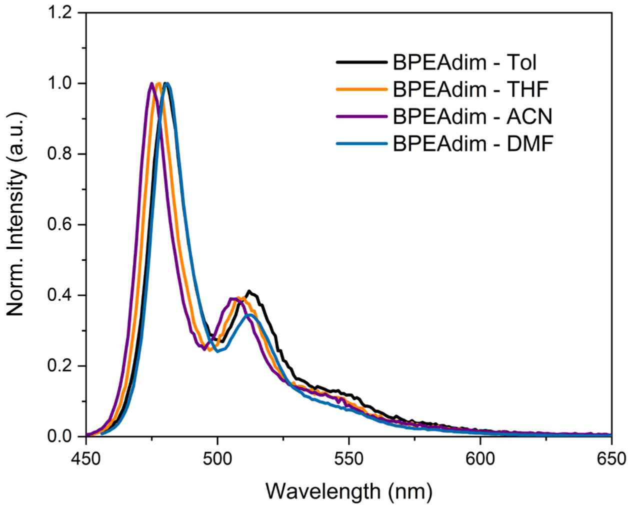

Figure S15. Normalized steady state emission of BPEAdim in toluene, THF, ACN, and DMF (excitation at 405 nm).

5.2 Time-resolved emission

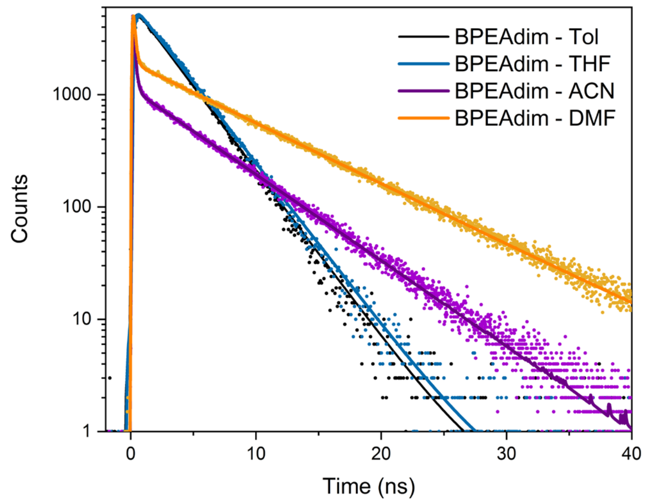

Figure S16. Time-resolved emission of BPEAdim in toluene, THF, ACN, and DMF (~10 µM) obtained with an excitation wavelength of 405 nm monitored at 480 nm. The fitting results are presented in Table S3.

Table S3. Emission lifetime of low concentrated solutions (~10 µM), BPEAdim in various solvents monitored at ~480 nm.

Solvent	τ (ns)
Toluene	2.9
THF	3.0
DMF	<0.1 (92%)[a] 7.9 (8%)
ACN	<0.1 (94%)[a] 5.6 (6%)

[a] Lifetime is within the IRF of the TCSPC setup.

5.3 fsTA

Table S4. Rate constants for BPEAdim in THF using the kinetic model in Figure 3b.

$1/k_1$ (ps)	$1/k_2$ (ps)
8.9	3220

Table S5. Rate constants for BPEAdim in ACN using the kinetic model in Figure 4d.

$1/k_1$ (ps)	$1/k_2$ (ps)	$1/k_3$ (ps)	$1/k_4$ (ns)	$1/k_5$ (ns)
5.3	63	6440	54	>60

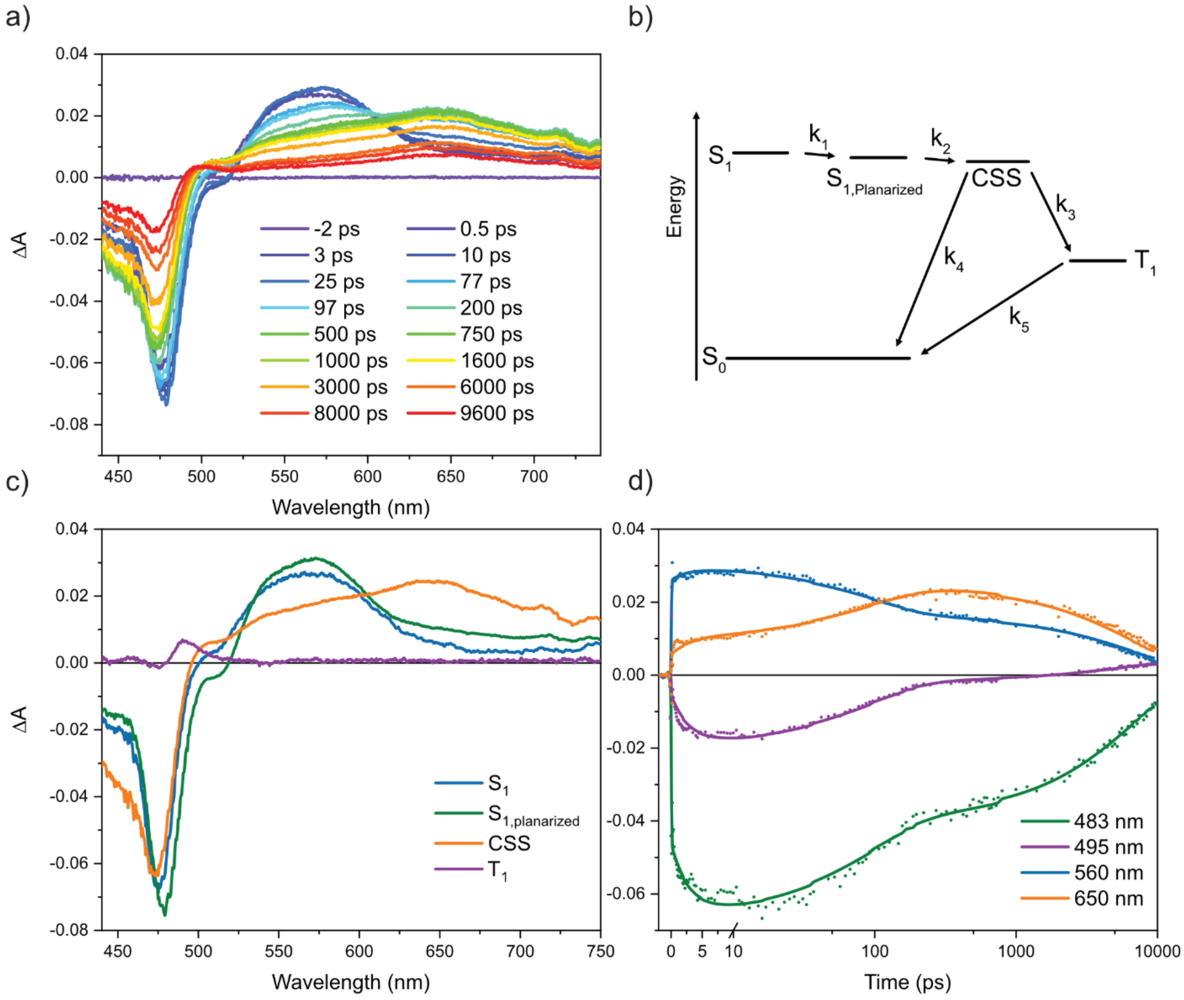

Figure S17. a) fsTA of a dilute solution of BPEAdim in DMF with excitation at 420 nm. b) Schematic representation of the kinetic model. c) Species associated spectra of the SVD analysis using the model shown in b). d) Selected kinetics of ground-state bleach at 483 nm, emission and triplet absorption at 495 nm, S_1 at 580 nm, and the CSS at 650 nm with the model data shown as a solid line. Note that the sensitized triplet spectrum from Figure S28 was used as the T_1 spectral component in the model, but with a scaling parameter to adjust its total amplitude.

Table S6. Rate constants for BPEAdim in DMF using the kinetic model in Figure S17b.

$1/k_1$ (ps)	$1/k_2$ (ps)	$1/k_3$ (ps)	$1/k_4$ (ns)	$1/k_5$ (ns)
3.0	97	8000	53	>60

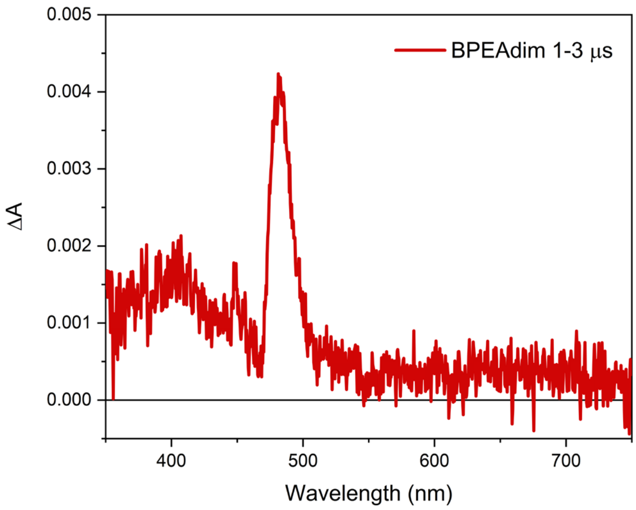

Figure S18. nsTA spectrum of BPEAdim in ACN following excitation with a pump pulse at 420 nm and integrating from 1 to 3 μs after the pump pulse.

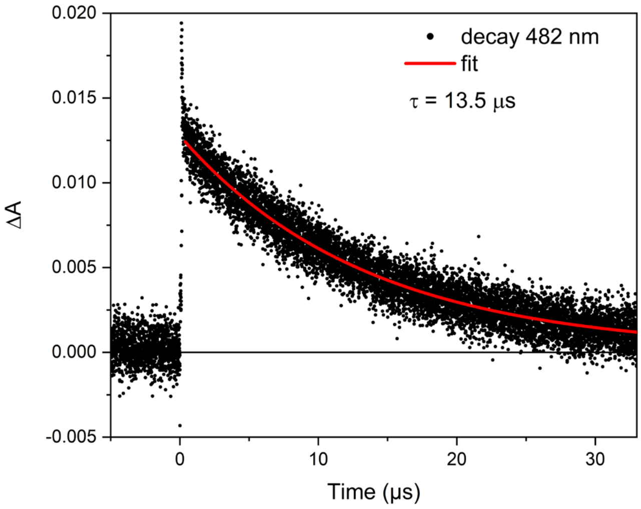

Figure S19. nsTA kinetic trace at 482 nm of BPEAdim in ACN following excitation with a pump pulse at 420 nm.

6 Photophysical characterization of BPEAtri

6.1 Steady-state absorption and emission

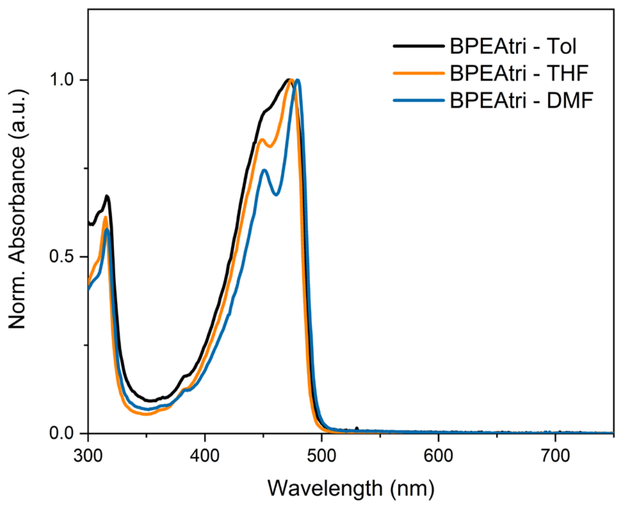

Figure S20. Normalized steady-state absorption of BPEAtri in toluene, THF, and DMF.

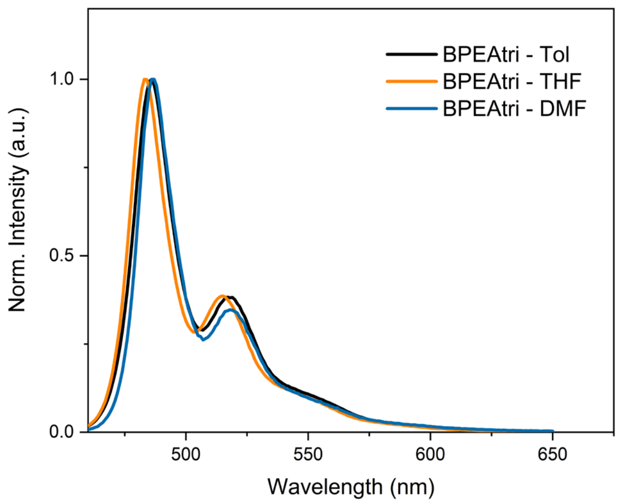

Figure S21. Normalized steady state emission of BPEAtri in toluene, THF, and DMF (excitation at 405 nm).

6.2 Time-resolved emission

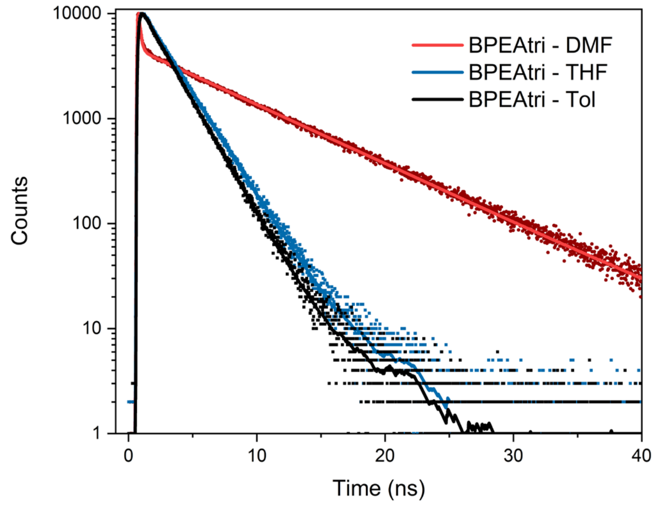

Figure S22. Time-resolved emission of BPEAtri in DMF, THF, and toluene (~10 µM) obtained with an excitation wavelength of 405 nm and monitored at 480 nm. The fitting results are presented in Table S7.

Table S7. Emission lifetime of low concentrated solutions (~10µM) of BPEAtri in various solvents monitored at 480 nm.

Solvent	τ (ns)
Toluene	2.0
THF	2.2
DMF	<0.1 (93%)[a] 7.6 (7%)

[a] Lifetime is within the IRF of the TCSPC setup.

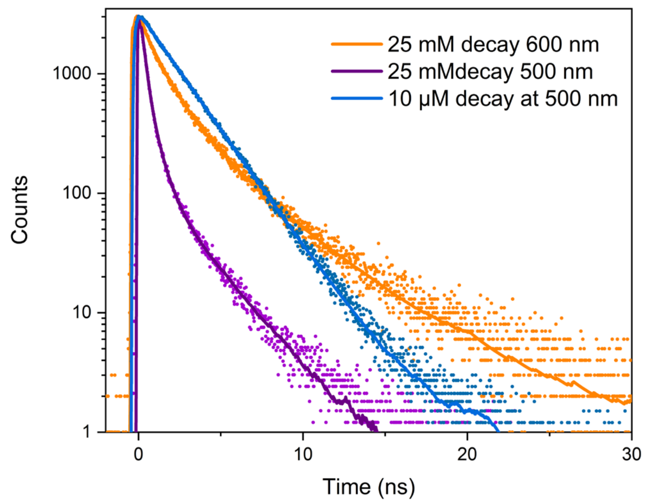

Figure S23. Time-resolved emission of BPEAtri in THF at high (25 mM) and low (10 µM) concentration obtained with an excitation wavelength of 405 nm and monitored at 500 and 600 nm. The fitting results are presented in Table S8.

Table S8. Emission lifetime of BPEAtri in THF at low (10 μM) and high (25 mM) concentrations monitored at 500 and 600 nm.

BPEAtri	500 nm	600 nm
C (mM)	τ (ns)	τ (ns)
10 μM	2.2	2.2
	0.18 (77%)	1.1 (73%)
25 mM	0.6 (20%)	3.0 (24%)
	2.5 (3%)	5.9 (3%)

6.3 fsTA

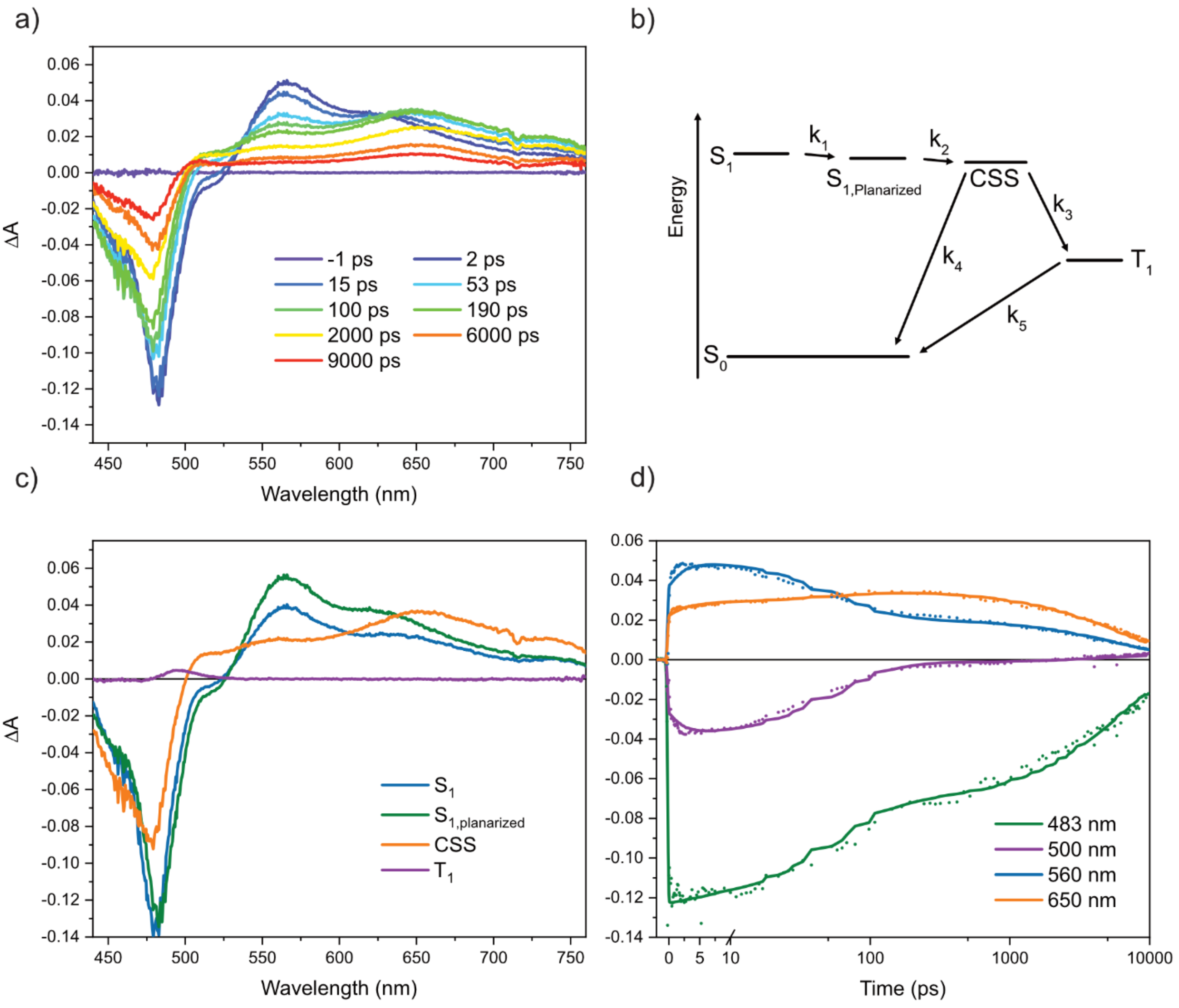

Figure S24. a) fsTA of a dilute solution of BPEAtri in DMF with excitation at 420 nm. b) Schematic representation of the kinetic model. c) Species associated spectra of the SVD analysis using the model shown in b. d) Selected kinetics of ground-state bleach at 483 nm, emission and triplet absorption at 495 nm, S_1 at 580 nm and the CSS at 650 nm with the model data shown as a solid line. Note that the sensitized triplet spectrum from Figure S29 as used as the T_1 spectral component in the model, but with a scaling parameter.

Table S9. Rate constants for BPEAtri in DMF using the kinetic model in Figure S24b.

$1/k_1$ (ps)	$1/k_2$ (ps)	$1/k_3$ (ps)	$1/k_4$ (ns)	$1/k_5$ (ns)
3.0	70	8000	53	>60

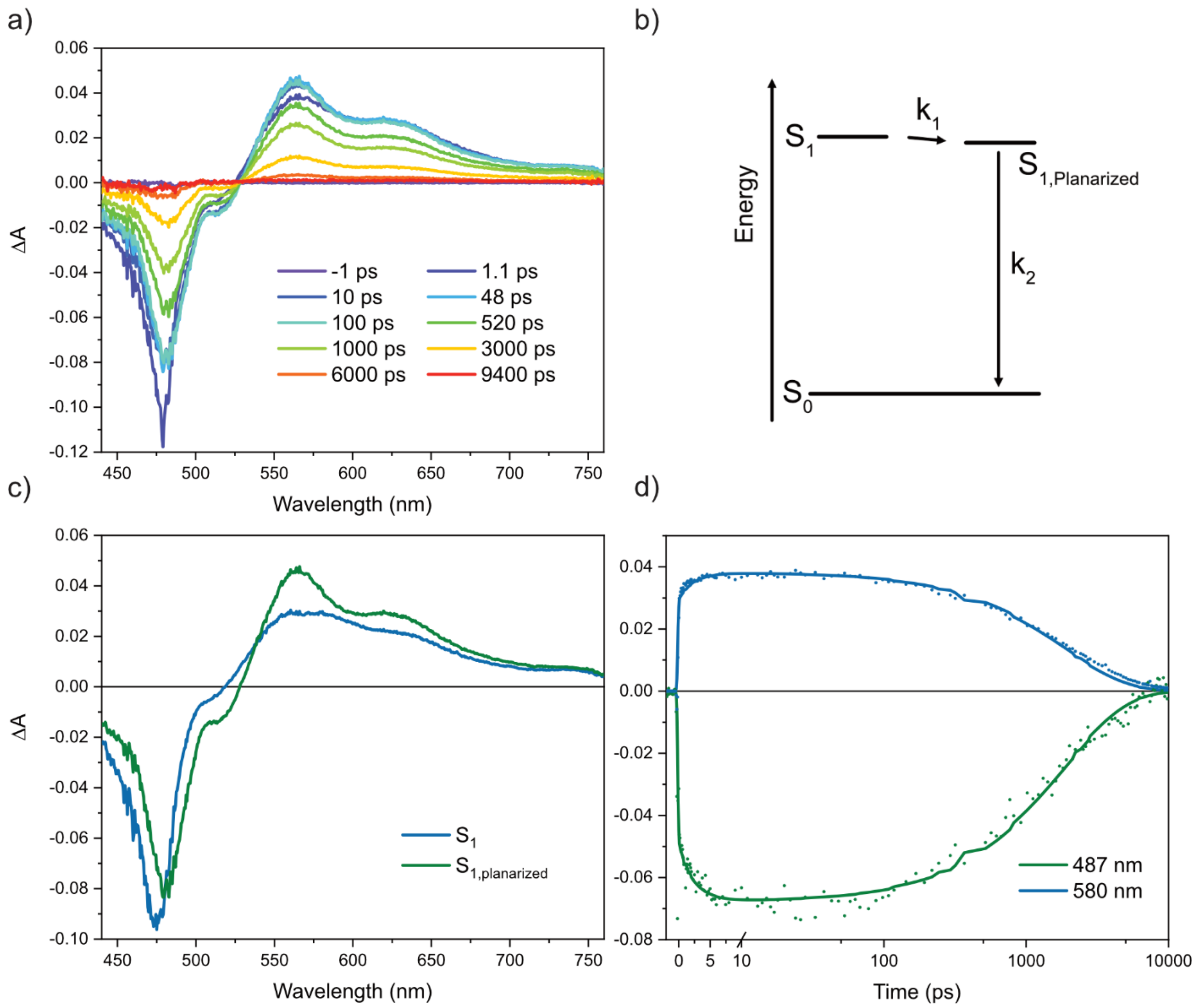

Figure S25. a) fsTA of a dilute solution of BPEAtri in THF with excitation at 420 nm. b) Schematic representation of the kinetic model. c) Species associated spectra of the SVD analysis using the model shown in b). d) Selected kinetics of ground-state bleach at 487 nm and S_1 at 580 nm with the model data shown as a solid line.

Table S10. Rate constants for BPEAtri in THF using the kinetic model in Figure S25b.

$1/k_1$ (ps)	$1/k_2$ (ps)
2.4	1960

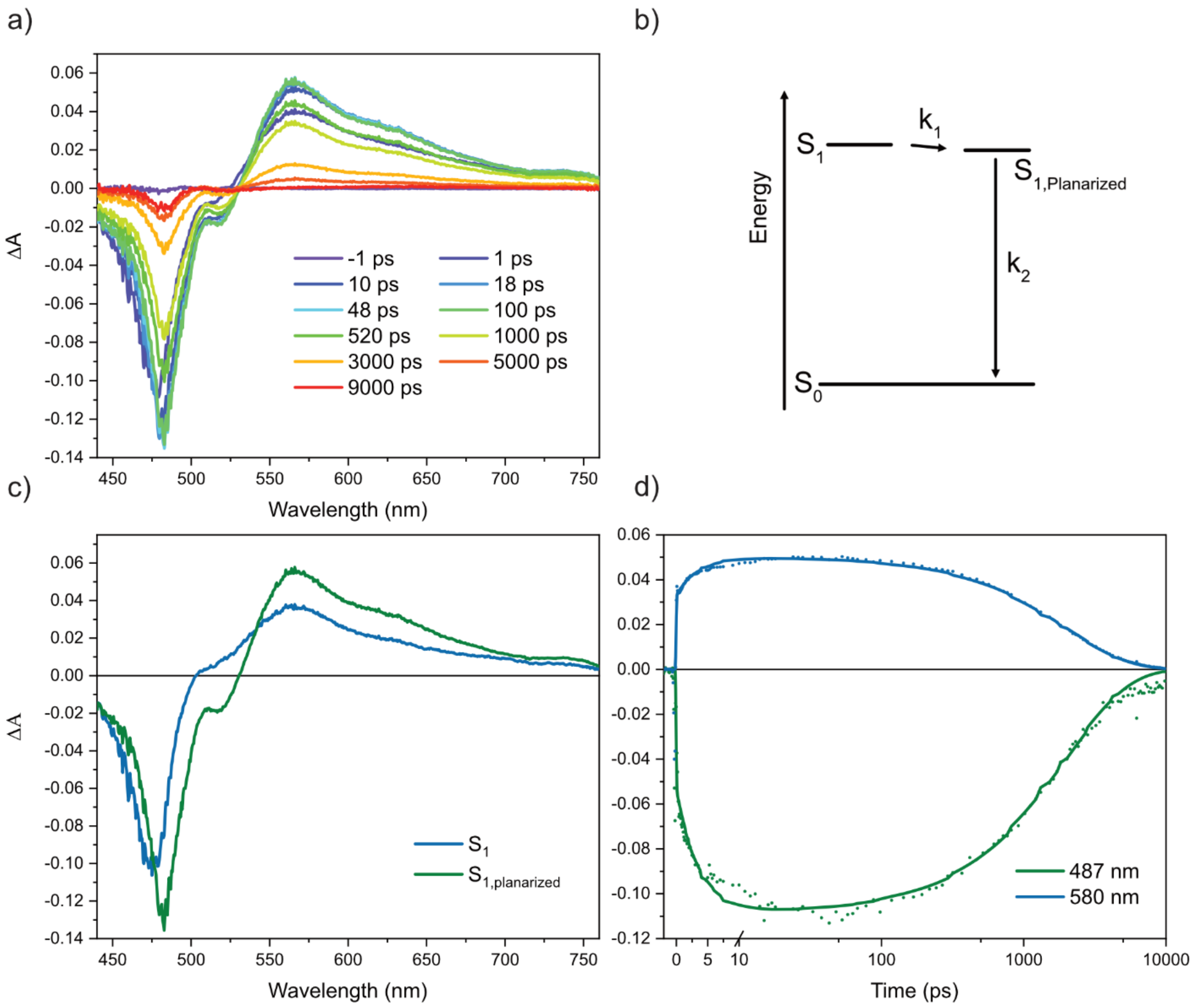

Figure S26. a) fsTA of a dilute solution of BPEAtri in toluene with excitation at 420 nm. b) Schematic representation of the kinetic model. c) Species associated spectra of the SVD analysis using the model shown in b). d) Selected kinetics of ground state bleach at 487 and S_1 at 580 nm with the model data shown as a solid line.

Table S11. Rate constants for BPEAtri in toluene using the kinetic model in Figure S26b.

$1/k_1$ (ps)	$1/k_2$ (ps)
3.6	2110

6.4 fsTA at high concentration

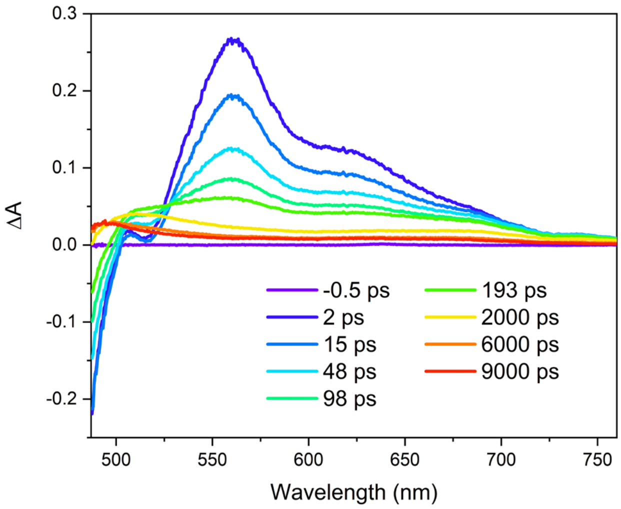

Figure S27. fsTA spectra of a 25 mM solution of BPEAtri in THF. Excited-state absorption at later time delays centered at 495 nm matches well with that of the triplet sensitized spectrum (Figure S29). Notably, the triplet excited state is formed without prior formation of the CSS state.

7 Triplet sensitization

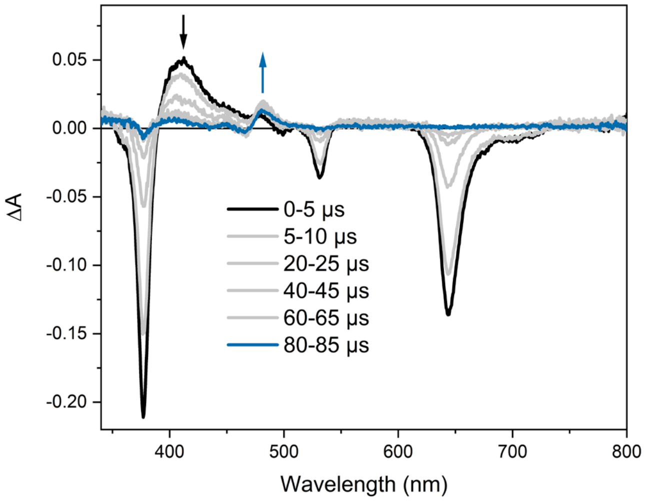

Figure S28. Triplet sensitization of BPEAdim. The nsTA spectra were obtained with a pump pulse at 536 nm in toluene exciting platinum octaethylporphyrin (PtOEP, 17 μM) followed by triplet energy transfer to BPEAdim (100 μM).

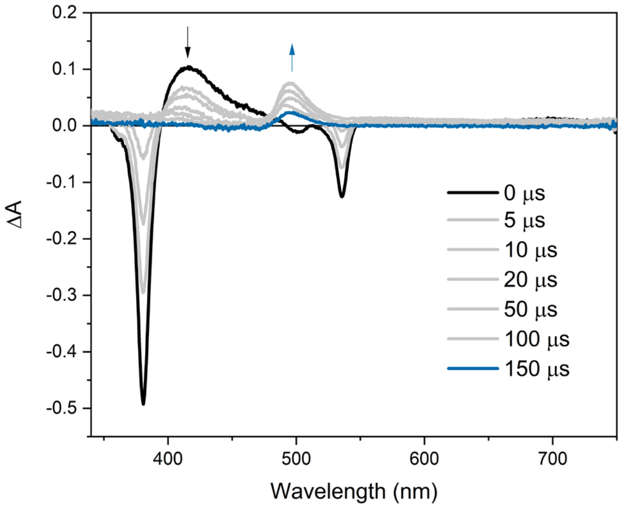

Figure S29. Triplet sensitization of BPEAtri. The nsTA spectra were obtained with a pump pulse at 536 nm in toluene exciting platinum octaethylporphyrin (PtOEP, 10 μM) followed by triplet energy transfer to BPEAtri (500 μM).

8 Synthetic details

General Experimental

All reagents and solvents were obtained from commercial suppliers and used as received unless otherwise stated. Anhydrous solvents were obtained using a solvent purification system from LC technology solutions Inc. All solvent ratios are volume/volume (v/v). Purification by flash column chromatography was carried out on silica gel (SiO_2, 60 Å, 40−63 µm). Thin-layer chromatography (TLC) was carried out using commercially available aluminum sheets precoated with silica gel with a fluorescent indicator and visualized under UV light at 254 and/or 360 nm. ^1H and $^{13}C\{^1H\}$ NMR spectra were recorded on a 500 MHz Agilent/Varian spectrometer equipped with a cold probe at 500 MHz and 126 MHz, respectively. ^1H NMR spectra were also recorded on a 500 MHz or 700 MHz Agilent/Varian instrument. Chemical shift values are reported in ppm and coupling constants (J) in Hz are reported as observed (±0.5 Hz). For simplicity, the coupling constants of signals for *para*-substituted aryl groups have been reported as pseudo first-order, even though they are second-order (AA'BB') spin systems. ^1H and $^{13}C\{^1H\}$ NMR spectra are referenced against the residual solvent peak ($CDCl_3$ δ_H 7.26, δ_C 77.16 ppm). FTIR spectra were recorded on a Thermo Nicolet 8700 FTIR spectrometer and samples were measured as cast films on salt plates (KBr). Matrix assisted laser desorption ionization (MALDI) High-resolution mass spectrometry (HRMS) spectra were recorded on a Bruker 9.4T Apex-Qe FTICR spectrometer. Electrospray ionization (ESI) HRMS were obtained from an Agilent 6220 oaTOF spectrometer. Direct Analysis in Real Time (DART) HRMS with nanospray ionization (NSI) were obtained from an Orbitrap Exploris 240 spectrometer. Melting points were measured on a Thomas Hoover capillary melting point apparatus. UV−Vis absorption measurements were done on a Cary 400 scan spectrophotometer using a quartz cuvette with a 1 cm pathlength, and the neat solvent was used as baseline; sh = shoulder. Differential scanning calorimetry (DSC) measurements were measured on a Perkin Elmer Pyris 1 DSC or Mettler and Toledo Polymer DSC instruments. Melting points from DSC analysis are reported as the endothermic maxima, except in cases when the sample decomposed, in which case the onset temperature of the decomposition exothermic peak is reported, as well as the exothermic maxima corresponding to the decomposition. Thermogravametric analysis (TGA) was conducted using a Perkin Elmer Pyris 1 TGA. All thermal analyses were carried out under a flow of N_2 with a heating rate of 10 °C/min. Thermal decomposition temperatures measured by TGA (as sample weight loss) are reported as T_d in which the temperature listed corresponds to the intersection of the tangent lines of the baseline and the edge of the feature corresponding to the first significant weight loss.

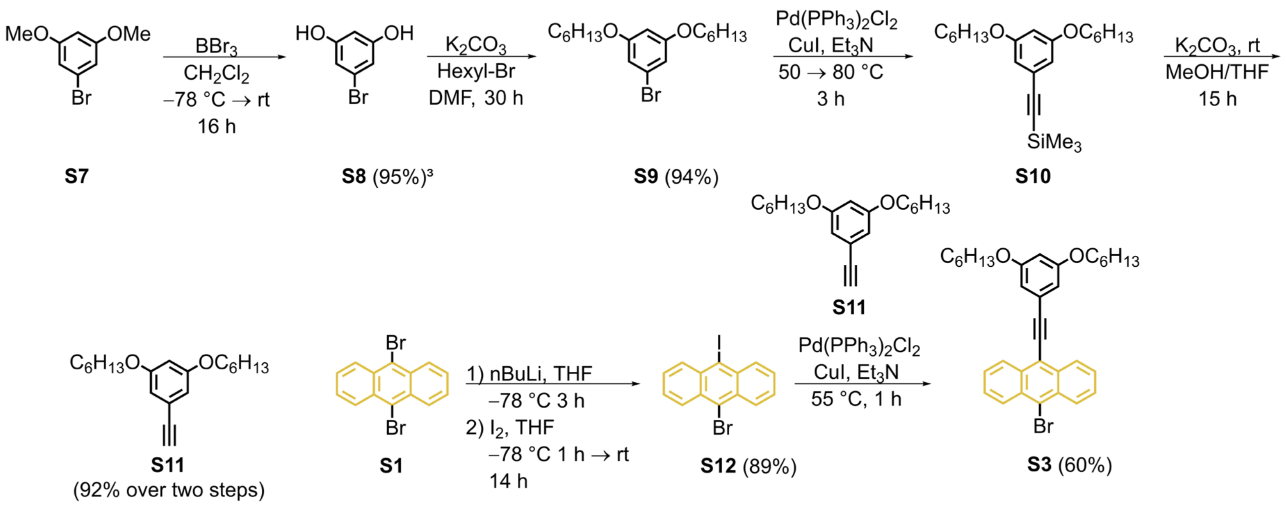

*Scheme S1. Synthesis of anthracene building block **S3**[5] from **S7**.*

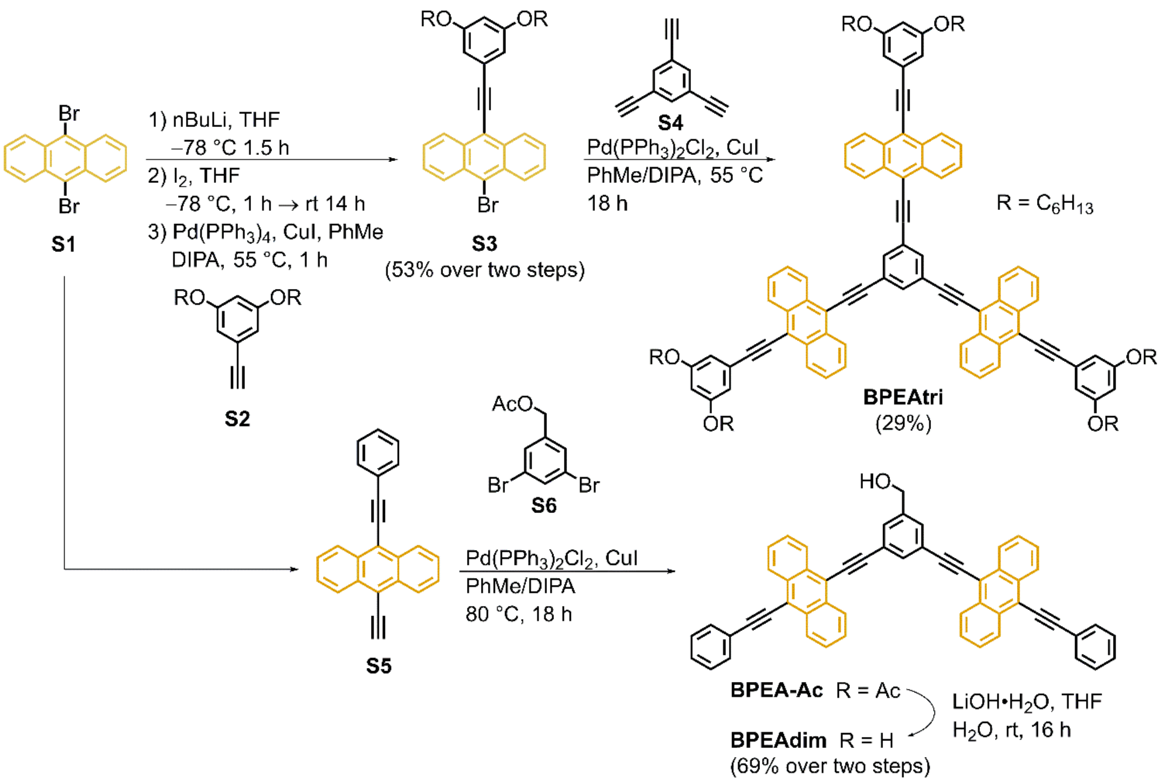

*Scheme S2. Synthesis of BPEAdim[6] and BPEAtri.[7] The synthesis of **S8–S11**,[8,9] and **S12**[7] were adapted from methods in literature.*

8.1 Experimental procedures

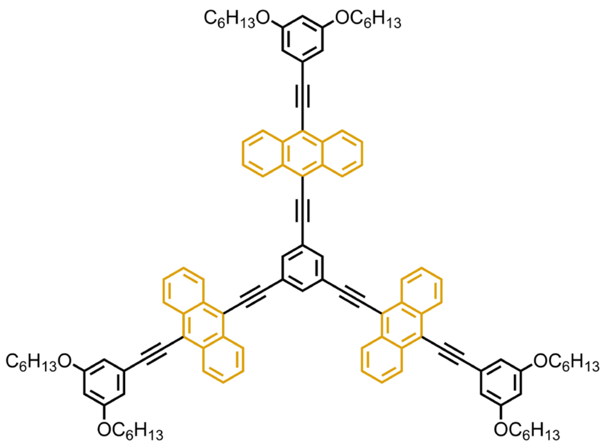

BPEAtri: A flask charged with **S3** (140 mg, 0.251 mmol), **S4** (11.4 mg, 0.076 mmol), $Pd(PPh_3)_2Cl_2$ (10.7 mg, 0.0152 mmol), and CuI (0.58 mg, 0.0030 mmol) was subjected to vacuum/N_2-purging (three cycles) before a degassed solution of DIPA/PhMe (1:2, 4 mL) was added. The reaction mixture was further sparged with N_2 for 10 min while stirring and heating to 55 °C. The reaction was stirred for 18 h at 55 °C, and then evaporated to dryness under a stream of N_2. The crude solid was dissolved in THF (5 mL) and passed through a celite plug and loaded on a BioBeads size exclusion column (THF). The first band was collected and was concentrated in vacuo. The crude material was subjected to flash column chromatography (SiO_2, CH_2Cl_2/hexanes 2:5 $\rightarrow$ 1:2). Precipitation from PhMe (3 mL) upon dilution with hexanes (10 mL), cooling the suspension to –30 °C for 6 h, and **BPEAtri** was isolated via suction filtration (42.2 mg, 35%) as an orange solid. Mp = 144–150 °C. R_f = 0.71 (silica gel, CH_2Cl_2/hexanes 1:1). IR (cast film CH_2Cl_2): 2926 (s), 2856 (m), 2193 (w), 1586 (s), 1171 (s) cm^{-1}. ^1H NMR (500 MHz, $CDCl_3$) δ 8.76 (d, J = 8.5 Hz, 6H), 8.70 (d, J = 8.4 Hz, 6H), 8.12 (s, 3H), 7.72 (t, J = 7.7 Hz, 6H), 7.67 (t, J = 7.9 Hz, 6H), 6.91 (d, J = 1.9 Hz, 6H), 6.55 (t, J = 2.0 Hz, 3H), 4.03 (t, J = 6.5 Hz, 12H), 1.83 (quint, J = 7.1 Hz, 12H), 1.51 (quint, J = 7.0 Hz, 12H), 1.42–1.34 (m, 24H), 0.94 (t, J = 6.9 Hz, 18H). $^{13}C\{^1H\}$ NMR (126 MHz, $CDCl_3$) δ 160.3, 134.3, 132.3, 132.1, 127.4, 127.19, 127.16, 126.9, 124.7, 124.5, 119.2, 117.7, 110.1, 103.0, 102.9, 100.7, 88.3, 85.9, 68.5, 31.8, 29.4, 25.9, 22.8, 14.2. MALDI HRMS (DCTB) m/z calcd for $C_{114}H_{114}O_6$ (M^+) 1578.8610, found 1578.8593.

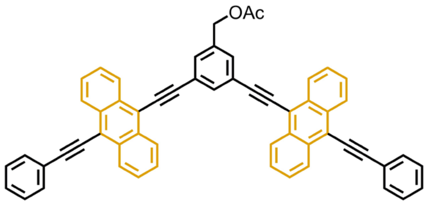

BPEA-Ac: A degassed solution of PhMe and DIPA (11 mL, 9:2) was added to a flask containing **S6** (50.0 mg, 0.162 mmol), **S5** (123 mg, 0.410 mmol), $Pd(PPh_3)_2Cl_2$ (11.4 mg, 0.0162 mmol), and CuI (0.77 mg, 0.0041 mmol). The mixture was heated to 80 °C and stirred for 18 h. The hot mixture was poured directly onto a silica gel column (PhMe, 15 cm x 5 cm) and the band at Rf = 0.47 (PhMe) was collected still partially mixed with homocoupled alkyne. The crude red solid (101 mg, 0.135 mmol, 83%) was used in the next step without further purification (≥90% purity by ^1H NMR spectroscopy). R_f = 0.47 (silica gel, PhMe). ^1H NMR (700 MHz, $CDCl_3$) δ 8.76–8.71 (m, 8H), 8.13 (t, J = 1.4 Hz, 1H), 7.82–7.79 (m, 6H), 7.75–7.66 (m, 8H), 7.65–7.40 (m, 6H) 5.27 (s, 2H), 2.23 (s, 3H). MALDI HRMS m/z calcd. for $C_{57}H_{34}O_2$ (M^+) 750.2553, found 750.2556.

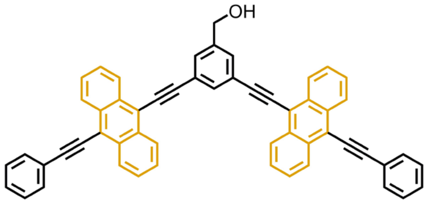

BPEAdim: Compound **S13** (78.0 mg, 0.104 mmol) was added to a solution of LiOH (26.2 mg, 0.623 mmol) in THF/H_2O (6 mL, 5:1), and the reaction was stirred for 16 h at rt. The reaction mixture was poured over H_2O (50 mL). The solid was collected by suction filtration, washed with satd. aq. $NaHCO_3$ (50 mL), H_2O (2 x 50 mL), and pentane (100 mL). The reddish-orange solid was purified by flash column chromatography (SiO_2, PhMe → 1:1 EtOAc/hexanes). The crude solid was dissolved in a minimal quantity of CH_2Cl_2 (10 mL) and precipitated upon addition of MeOH (60 mL). The orange precipitate **BPEAdim** (50.3 mg, 68%) was collected via suction filtration pure after washing with pentane (3 x 5 mL) and drying under vaccum. R_f = 0.16 (silica gel, PhMe). Mp = no melt was observed ≤ 300 °C (272–274 °C color change from orange to dark brown). IR (cast film CH_2Cl_2) 3265 (br w), 3080 (w), 3058 (w), 3033 (w), 1588 (w), 763 (s), 751 (m) cm^{-1}. ^1H NMR (500 MHz, $CDCl_3$) δ 8.76 (t, J = 8.8 Hz, 8H), 8.11 (t, J = 1.5 Hz, 1H), 7.85 (s, 2H), 7.82–7.78 (d, J = 6.7 Hz, 4H), 7.69 (dq, J = 1.9, 8.0 Hz, 8H), 7.50–7.42 (m, 6H), 4.91 (d, J = 6.0 Hz, 2H), 1.89 (t, J = 6.0 Hz, 1H). ^{13}C NMR (126 MHz, DMSO-d_6) δ 131.7, 131.5, 131.3, 130.4, 129.5, 129.0, 128.0, 127.9, 127.0, 126.8, 122.8, 122.1, 117.9, 117.3, 103.0, 101.9, 86.4, 85.8, 62.1 (two signals coincident or not observed). MALDI HRMS *m/z* calcd. for $C_{55}H_{32}O$ (M^+) 708.2453, found 708.2447. DSC: decomposition, 280 (onset), 282 °C (peak); 289 (onset), 295 °C (peak).

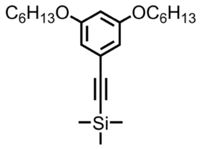

Compound S10: A flask charged with **S9** (2.00 g, 5.60 mmol), $Pd(PPh_3)_2Cl_2$ (197 mg, 0.280 mmol), and CuI (5.15 mg, 0.0271 mmol) was subjected to vacuum/N_2-purging (three cycles), and a degassed solution of Et_3N (25 mL) was then added. The reaction mixture was stirred while sparging with N_2 for 10 min, heated to 50 °C, and TMSA (1.37 g, 1.97 mL, 14.0 mmol) was added. The reaction was heated to 80 °C and stirred for 3 h. The reaction mixture was poured into satd. aq. NH_4Cl (100 mL) and extracted with CH_2Cl_2 (4 x 25 mL). The organic phase was washed with H_2O (50 mL) and brine (2 x 80 mL), dried over Na_2SO_4, filtered, and concentrated under reduced pressure. The crude material was subjected to flash column chromatography (silica gel, hexanes, CH_2Cl_2) to afford the intermediate **S10** (1.95 g, ≥90% purity by ^1H NMR spectroscopy) as a yellow oil. ^1H NMR (700 MHz, $CDCl_3$) δ 6.59 (d, J = 2.3 Hz, 2H), 6.43 (t, J = 2.3 Hz, 1H), 3.91 (t, J = 6.5 Hz, 4H), 1.76 (quint, J = 6.5 Hz, 4H), 1.42 (quint, J = 7.6 Hz, 4H), 1.34–1.29 (m, 8H), 0.90 (t, J = 6.9 Hz, 6H), 0.24 (s, 9H).

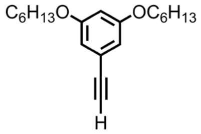

Compound S11: To a solution of **S10** (1.00 g, 2.67 mmol) in THF (100 mL) and MeOH (100 mL) was added, K_2CO_3 (1.11 g, 8.10 mmol). After stirring for 15 h at rt, the reaction mixture was diluted with H_2O (200 mL) and Et_2O (200 mL). The organic phase was separated, and the aqueous phase was extracted (60 mL), the combined organic phases were washed with H_2O (2 x 20 mL) and brine (2 x 100 mL), dried over Na_2SO_4, filtered, and concentrated under reduced pressure. The crude material was subjected to flash column chromatography (silica gel, 1:10 → 1:5 CH_2Cl_2/hexanes). Drying *in vacuo* to afforded compound **S11** (799 mg, 92% over two steps) as a pale-yellow oil. R_f = 0.22 (CH_2Cl_2/hexane 1:9). IR (cast film CH_2Cl_2): 3296 (w), 2956 (m), 2932 (m), 2872 (m), 2860 (m), 1589 (s), 1171 (s) cm^{-1}. ^1H NMR (700 MHz, $CDCl_3$) δ 6.62 (d, J = 2.1 Hz, 2H), 6.46 (t, J = 2.1 Hz, 1H), 3.92 (t, J = 7.0 Hz, 4H), 3.01 (s, 1H), 1.76 (quint, J = 7.0 Hz, 4H), 1.43 (quint, J = 7.0 Hz, 4H), 1.35–1.31 (m, 8H), 0.90 (t, J = 7.0 Hz, 6H). ^{13}C NMR (126 MHz, $CDCl_3$) δ 160.2, 123.3, 110.6, 103.3, 83.9,

76.6, 68.3, 31.7, 29.3, 25.8, 22.7, 14.2. DART-NSI+ HRMS *m/z* calcd for $C_{20}H_{31}O_2$ ($[M+H]^+$) 303.2319, found 303.2317.

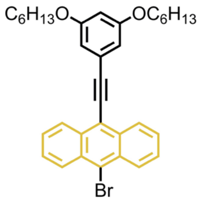

Compound S3: A flask charged with **S12** (443 mg, 1.16 mmol), **S11** (350 mg, 1.16 mmol), $Pd(PPh_3)_4$ (26.8 mg, 0.0232 mmol), and CuI (4.42 mg, 0.0232 mmol) was subjected to vacuum/N_2-purging (three cycles) before a degassed solution of DIPA/PhMe (1:4, 8 mL) was added. The reaction was stirred while heating at 55 °C for 1 h and then cooled to –30 °C and diluted with hexanes (15 mL). After 20 min, the resulting precipitate was collected by suction filtration while cold. The crude material was subjected to flash column chromatography (silica gel, CH_2Cl_2/hexanes 1:10 → 3:10). The resulting product was dissolved in hot PhMe (3 mL), cooled to rt, diluted with hexanes (20 mL), and the resulting mixture was cooled to –30 °C. Suction filtration afforded **S3** (450 mg, 70%) as a yellow solid. Mp = 98–100 °C. R_f = 0.22 (SiO_2, CH_2Cl_2/hexanes 1:3). IR (cast film CH_2Cl_2): 3068 (w), 3051 (w), 2953 (m), 2929 (m), 2869 (m), 2855 (m), 1578 (s), 1314 (s), 1167 (s) cm^{-1}. ^1H NMR (500 MHz, $CDCl_3$) δ 8.71–8.66 (m, 2H), 8.60–8.55 (m, 2H), 7.67–7.61 (m, 4H), 6.89 (d, *J* = 2.2 Hz, 2H), 6.55 (t, *J* = 2.3 Hz, 1H), 4.03 (t, *J* = 6.5 Hz, 4H), 1.82 (quint, *J* = 6.6 Hz, 4H), 1.50 (quint, *J* = 7.3 Hz, 4H), 1.41–1.33 (m, 8H), 0.93 (t, *J* = 7.0 Hz, 6H). ^{13}C NMR (126 MHz, $CDCl_3$) δ 160.5, 133.2, 130.4, 128.4, 127.6, 127.4, 127.0, 124.6, 124.3, 118.4, 110.2, 103.0, 102.2, 85.4, 68.5, 31.7, 29.4, 25.9, 22.8, 14.2. MALDI HRMS (DCTB) *m/z* calcd for $C_{34}H_{37}BrO_2$ (M^+) 556.1971, found 556.1970. TGA: Td ≈ 370 °C. DSC: Mp = 99.8 °C (onset), 100 °C (peak), decomposition, 278 °C (onset), 297 °C (peak).

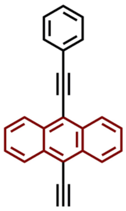

Compound S5: This building block can be prepared as previously reported.[7] Mp = no melt was observed ≤ 300 °C (slowly goes dark brown upon heating to 150 °C). ^1H NMR (700 MHz, $CDCl_3$) δ 8.71–8.68 (m, 2H), 8.68–8.61 (m, 2H), 7.79–7.77 (m, 2H), 7.66–7.60 (m, 4H), 7.48 (m, 4H), 4.08 (s, 1H).

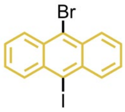

Compound S12: Prepared as previously reported.[7] Mp = 216–219 °C. ^1H NMR (700 MHz, $CDCl_3$) δ 8.59–8.55 (m, 4H), 7.65–7.58 (m, 4H). ^1H NMR spectral characteristics are consistent with those previously reported.[7]

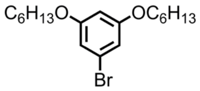

Compound S9: To a mixture of K_2CO_3 (8.77g, 63.5 mmol), and **S8** (4.0 g, 21.2 mmol) in DMF (190 mL) was added, 1-bromohexane (7.3 g, 6.3 mL, 44.4 mmol) and the mixture was heated to reflux for 30 h with vigorous stir. The reaction was cooled to rt, Et_2O (100 mL) and H_2O (100 mL) were added, and the phases separated. The aqueous phase was extracted (2 x 100 mL) and the combined extract was washed with brine (5 x 50 mL), dried over $MgSO_4$, filtered, and concentrated to an oil. Column chromatography (silica gel, hexanes) gave product (7.1 g, 94%) as a clear colourless oil. R_f = 0.56 (SiO_2, hexanes). IR (cast film CH_2Cl_2): 2955 (s), 2932 (s), 2871 (m), 2859 (m), 1598 (s), 1576 (s), 1169 (s) cm^{-1}. ^1H NMR (700 MHz, $CDCl_3$) δ 6.64 (d, *J* = 2.0 Hz, 2H), 6.37

(t, J = 2.0 Hz, 1H), 3.90 (t, J = 6.5 Hz, 4H), 1.75 (quint, J = 7.0 Hz, 4H), 1.44 (quint, J = 6.5 Hz, 4H), 1.35–1.30 (m, 8H), 0.91 (t, J = 7.0 Hz, 6H). ^{13}C NMR (126 MHz, $CDCl_3$) 160.9, 123.0, 110.4, 100.8, 68.5, 31.7, 29.2, 25.8, 22.7, 14.2. ^{1}H NMR spectral characteristics are consistent with homologous compounds previously reported.[11] DART-NSI+ HRMS m/z calcd for $C_{18}H_{30}BrO_2$ ($[M+H]^+$) 357.1424, found 357.1427.

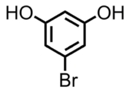

Compound S8: Prepared using a modified procedure as previously reported.[12] Mp = 76–78 °C.^{1}H NMR (500 MHz, DMSO-d_6) δ 9.66 (s, 2H), 6.38 (d, J = 2.1 Hz, 2H), 6.19 (t, J = 2.1 Hz, 1H). ^{1}H NMR spectral characteristics are consistent with those previously reported.[10] ESI HRMS m/z calcd for $C_6H_4BrO_2$ ($[M - H]^-$) 186.9400, found 186.9403.

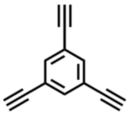

Compound S4: Prepared as previously reported.[12] ^{1}H NMR (700 MHz, $CDCl_3$) δ 7.57 (s, 3H), 3.10 (s, 3H). ^{1}H NMR spectral characteristics are consistent with those previously reported.[12]

8.2 Experimental data and compound characterization

Analysis Info

Analysis Name:	U:\Service\230228_0_A3_000001.d	Acquisition Date:	2/28/2023 3:40:20 PM
Method:	MALDI_DCTB_1k_new	Instrument:	apex-Qe
Sample Name:	ZWS-6-66	Operator:	
Comment:	Z. Schroeder, R. Tykwinski, DCTB as matrix, 9.4T FTICR MS		

Acquisition Parameter

Ionisation Mode: Positive MALDI Mode: n/a

Generate Molecular Formula Parameter

Formula, min.:	C93							
Formula, max.:	N0O10							
Measured m/z:	1578.86			Charge:	1	Tolerance:	2	mDa
Check Valence:	no	Min.:	0	Max.:	0			
Nitrogen Rule:	no							
Filter H/C Ratio:	no	Min.:	0	Max.:	3			
Estimate # of C:	yes					Electron Configuration:	both	

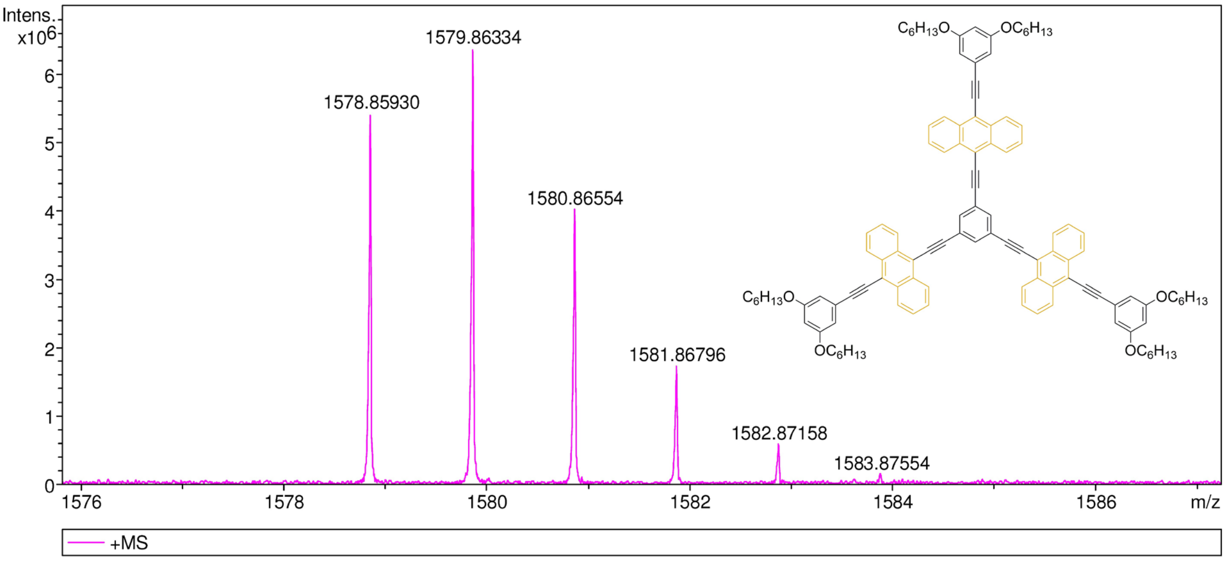

Sum Formula	Sigma	m/z	Err [ppm]	Mean Err [ppm]	Err [mDa]	rdb	N Rule	e⁻
C 114 H 114 O 6	0.021	1578.86099	1.07	1.10	1.69	58.00	ok	odd

*Figure S30. MALDI HRMS analysis of **BPEAtri** (matrix: DCTB).*

Analysis Info

Analysis Name:	T:\Service\220325_0_J8_000001.d	Acquisition Date:	25/03/2022 10:16:23 AM
Method:	MALDI_DCTB_mz600_new	Instrument:	apex-Qe
Sample Name:	ZWS-5-14-B	Operator:	
Comment:	Z. Schroeder, R. Tykwinski, DCTB as matrix, 9.4T FTICR MS		

Acquisition Parameter

Ionisation Mode:	Positive	MALDI Mode:	n/a

Generate Molecular Formula Parameter

Formula, min.:	C47						
Formula, max.:	N0						
Measured m/z:	750.256			Charge:	1	Tolerance:	2 mDa
Check Valence:	no	Min.:	0	Max.:	0		
Nitrogen Rule:	no						
Filter H/C Ratio:	no	Min.:	0	Max.:	3		
Estimate # of C:	yes					Electron Configuration:	both

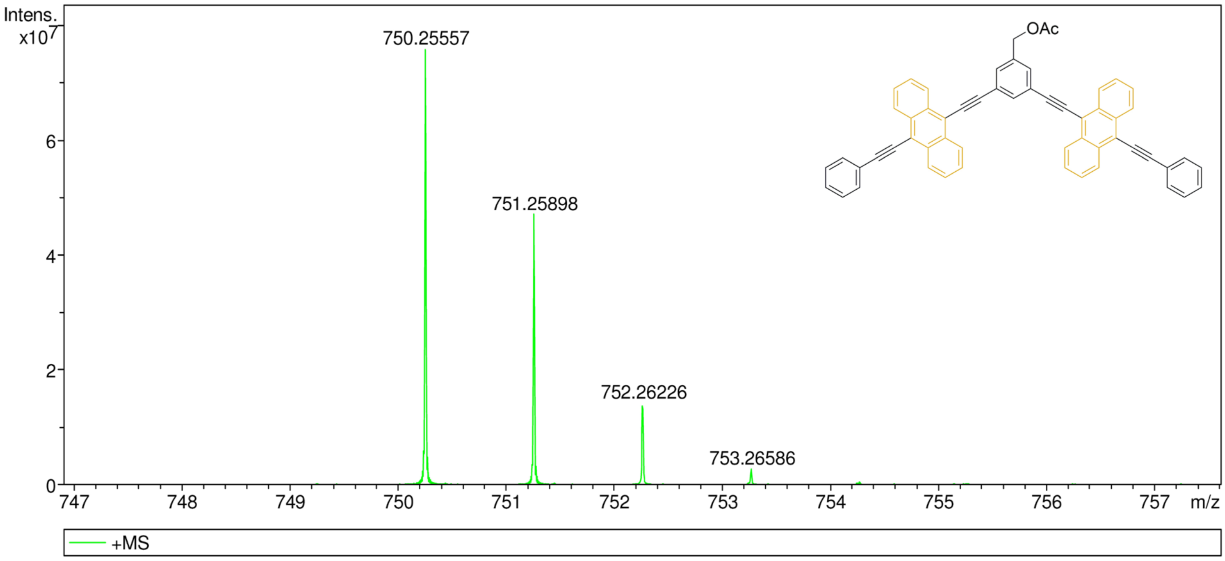

Sum Formula	Sigma	m/z	Err [ppm]	Mean Err [ppm]	Err [mDa]	rdb	N Rule	e⁻
C 57 H 34 O 2	0.004	750.25533	-0.31	-0.34	-0.23	41.00	ok	odd

Figure S31. MALDI HRMS analysis of ***BPEA-Ac*** *(matrix: DCTB).*

Analysis Info

Analysis Name:	T:\Service\220414_0_A13_000001.d	Acquisition Date:	14/04/2022 3:54:14 PM
Method:	MALDI_DCTB_mz600_new	Instrument:	apex-Qe
Sample Name:	ZWS-5-22	Operator:	
Comment:	Z. Schroeder, R. Tykwinski, DCTB as matrix, 9.4T FTICR MS, neg ion		

Acquisition Parameter

Ionisation Mode: Positive MALDI Mode: n/a

Generate Molecular Formula Parameter

Formula, min.:	C44						
Formula, max.:	N0						
Measured m/z:	708.245			Charge:	1	Tolerance:	2 ppm
Check Valence:	no	Min.:	0	Max.:	0		
Nitrogen Rule:	no						
Filter H/C Ratio:	no	Min.:	0	Max.:	3		
Estimate # of C:	yes					Electron Configuration:	both

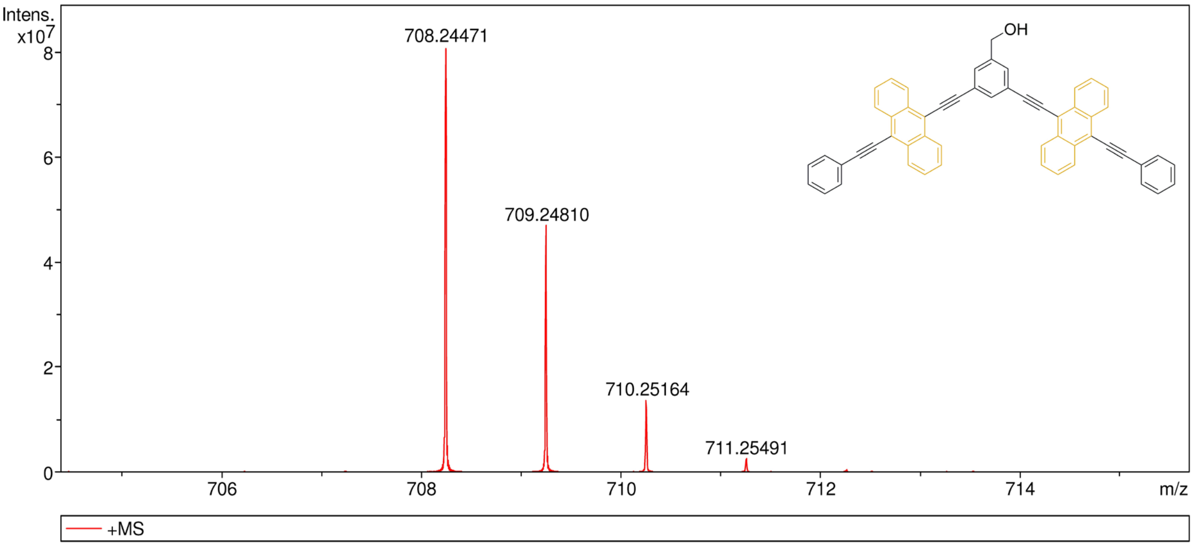

Sum Formula	Sigma	m/z	Err [ppm]	Mean Err [ppm]	Err [mDa]	rdb	N Rule	e⁻
C 55 H 32 O 1	0.010	708.24477	0.09	0.04	0.06	40.00	ok	odd

Figure S32. MALDI HRMS analysis of ***BPEAdim*** *(matrix: DCTB).*

Analysis Info

Analysis Name:	U:\Service\230215_0_E9_000001.d	Acquisition Date:	2/15/2023 1:12:41 PM
Method:	MALDI_DCTB_mz600_new	Instrument:	apex-Qe
Sample Name:	ZWS-6-47	Operator:	
Comment:	Z. Schroeder, R. Tykwinski, DCTB as matrix, 9.4T FTICR MS		

Acquisition Parameter

Ionisation Mode: Positive   MALDI Mode: n/a

Generate Molecular Formula Parameter

Formula, min.:	C26							
Formula, max.:	N0O4Br							
Measured m/z:	556.197			Charge:	1	Tolerance:	2	mDa
Check Valence:	no	Min.:	0	Max.:	0			
Nitrogen Rule:	no							
Filter H/C Ratio:	no	Min.:	0	Max.:	3			
Estimate # of C:	yes					Electron Configuration:	both	

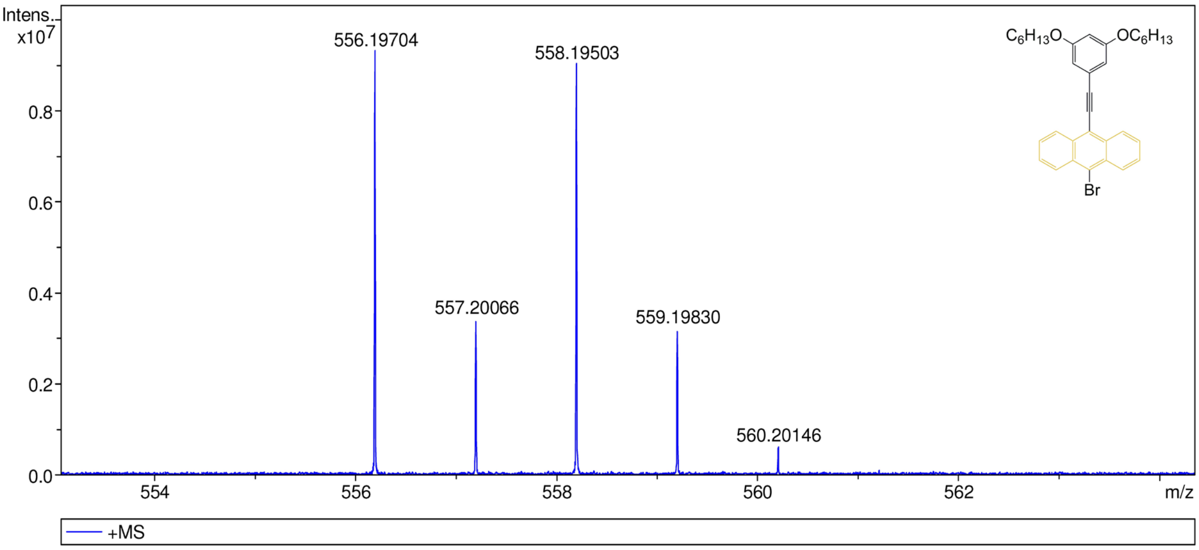

Sum Formula	Sigma	m/z	Err [ppm]	Mean Err [ppm]	Err [mDa]	rdb	N Rule	e⁻
C 34 H 37 Br 1 O 2	0.022	556.19714	0.19	0.56	0.11	16.00	ok	odd

*Figure S33. MALDI HRMS analysis of **S3** (matrix: DCTB).*

UNIVERSITY OF ALBERTA | **Mass Spectrometry Facility** | **+1-780-492-5577**

Sample	ZWS-6-40, Zachary Schroeder, Tykwinski, Dart			***File Name***	D:\DART\Data\Service\202302\23021603.raw
Comment	ZWS-6-40, Zachary Schroeder, Tykwinski, Dart			***Instrument Model***	Orbitrap Exploris 240
Ion Mode	NSI+	***Scan***	100	***Scan Filter***	FTMS + p NSI Full ms [100.0000-600.0000]
Scan Type	Full	***Spectrum Type***	MS		

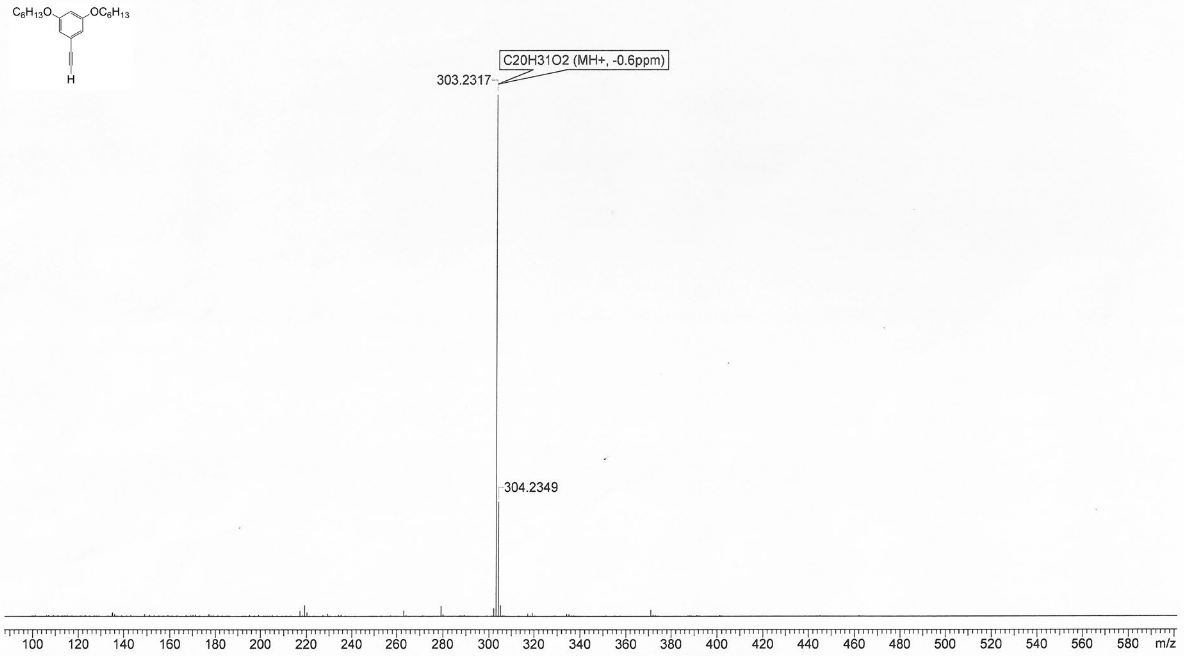

No.	Formula	Monoisotopic Mass	Error (ppm)	RDBE
1	C20H31O2	303.2319	-0.55	5.5

*Figure S34. DART NSI+ HRMS analysis of **S11**.*

UNIVERSITY OF ALBERTA — Mass Spectrometry Facility — +1-780-492-5577

**Sample**	ZWS-6-32, Zachary Schroeder, Tykwinski, Dart		
**Comment**	ZWS-6-32, Zachary Schroeder, Tykwinski, Dart		
**Ion Mode**	NSI+	**Scan**	132
**Scan Type**	Full	**Spectrum Type**	MS

**File Name**	D:\DART\Data\Service\202302\23021604.raw
**Instrument Model**	Orbitrap Exploris 240
**Scan Filter**	FTMS + p NSI Full ms [100.0000-600.0000]

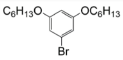

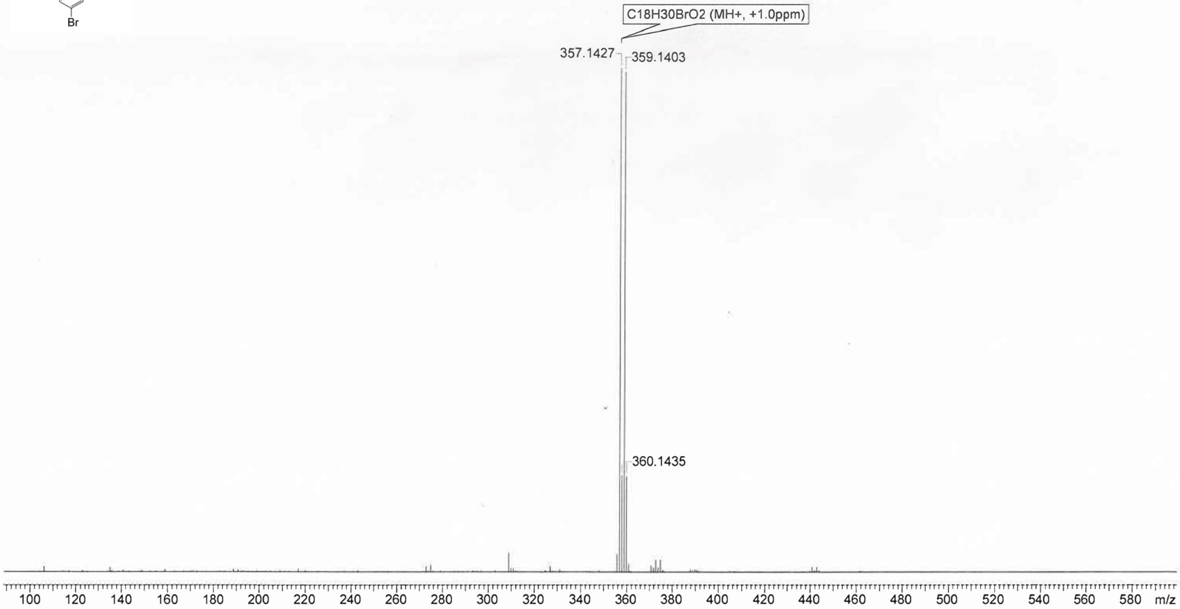

No.	Formula	Monoisotopic Mass	Error (ppm)	RDBE
1	C18H30BrO2	357.1424	1.03	3.5;4.5;5.5;6.5

Figure S35. DART NSI+ HRMS analysis of ***S9****.*

Qualitative Analysis Report

**Data Filename**	23021514.d	**Name**	Z. Schroeder, Tykwinski
**Sample Name**	zws 6 33	**Position**	-1
**Instrument Name**	oaTOF6220	**Operator**	ami
**Acq Method**		**DA Method**	ami_da.m

User Spectra

Fragmentor Voltage	Collision Energy	Ionization Mode
100	0	Esi

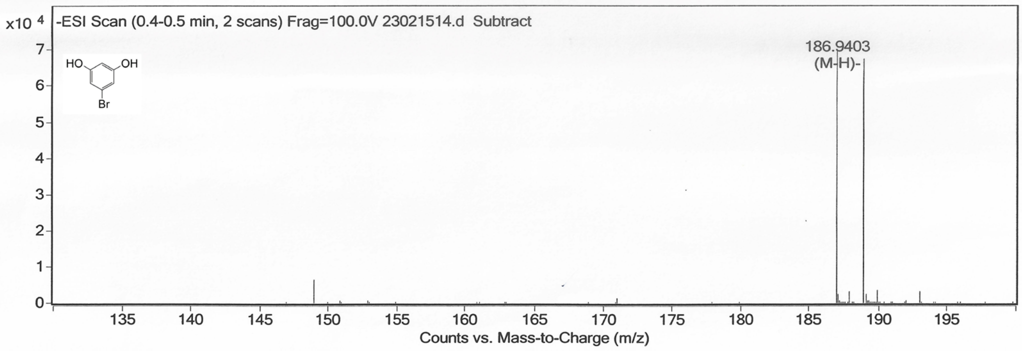

Formula Calculator Results

Formula	Ion Formula	Mass	Calc Mass	m/z	Calc. m/z	Diff (mDa)	Diff (ppm)	DBE	Ion Species	Score
C6 H5 Br O2	C6 H4 Br O2	187.9476	187.9473	186.9403	186.94	-0.29	-1.55	4	(M-H)-	98.81

--- End Of Report ---

Mass Spectrometry Facility, Dept. Of Chemistry
University of Alberta, 1-780-492-5577

Page 1 of 1

Printed at: 8:31 PM on: 2/15/2023

Figure S36. ESI HRMS analysis of ***S8****.*

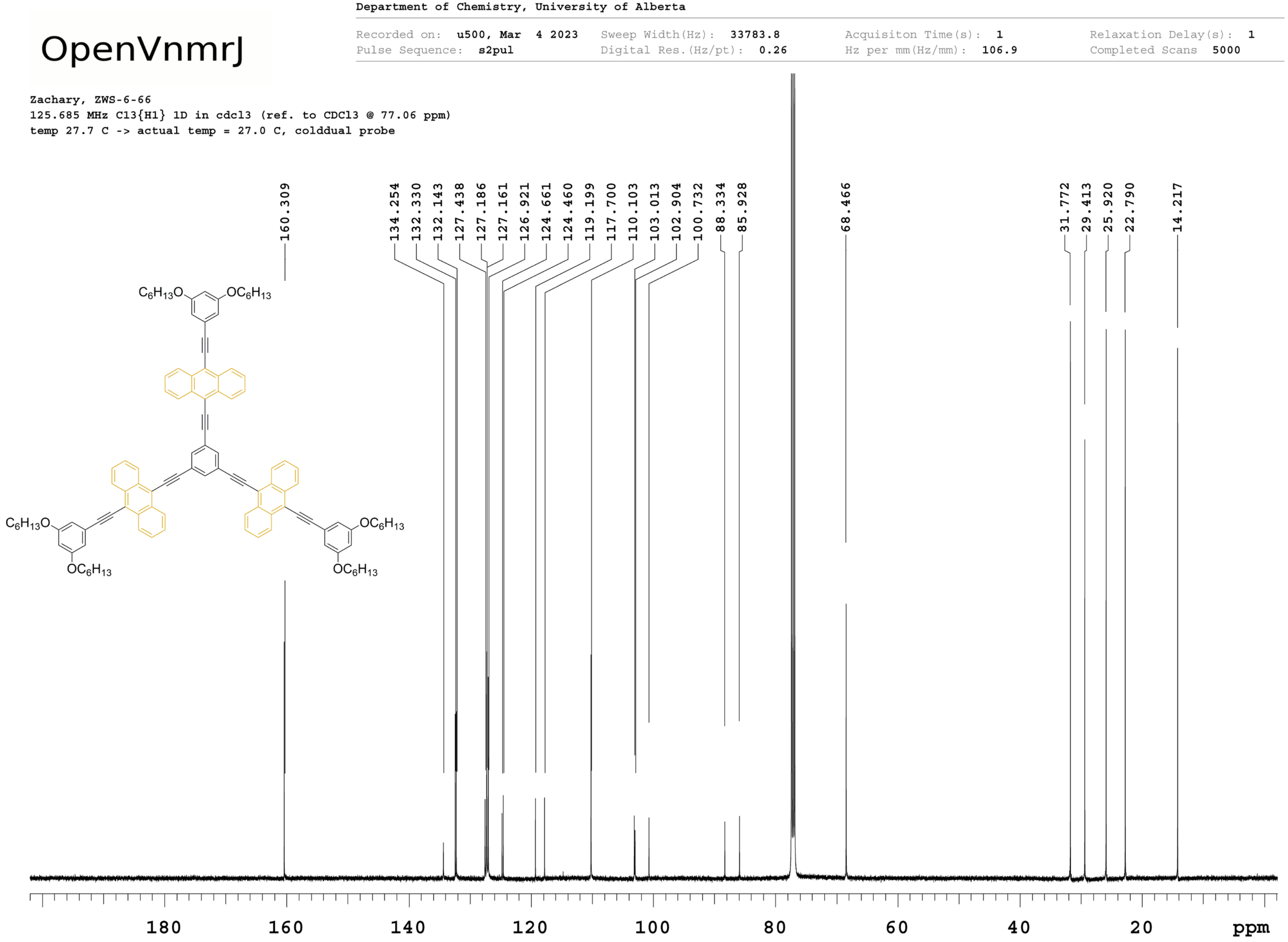

*Figure S37. $^{13}C\{^1H\}$ NMR spectrum (126 MHz) of **BPEAtri** recorded in $CDCl_3$.*

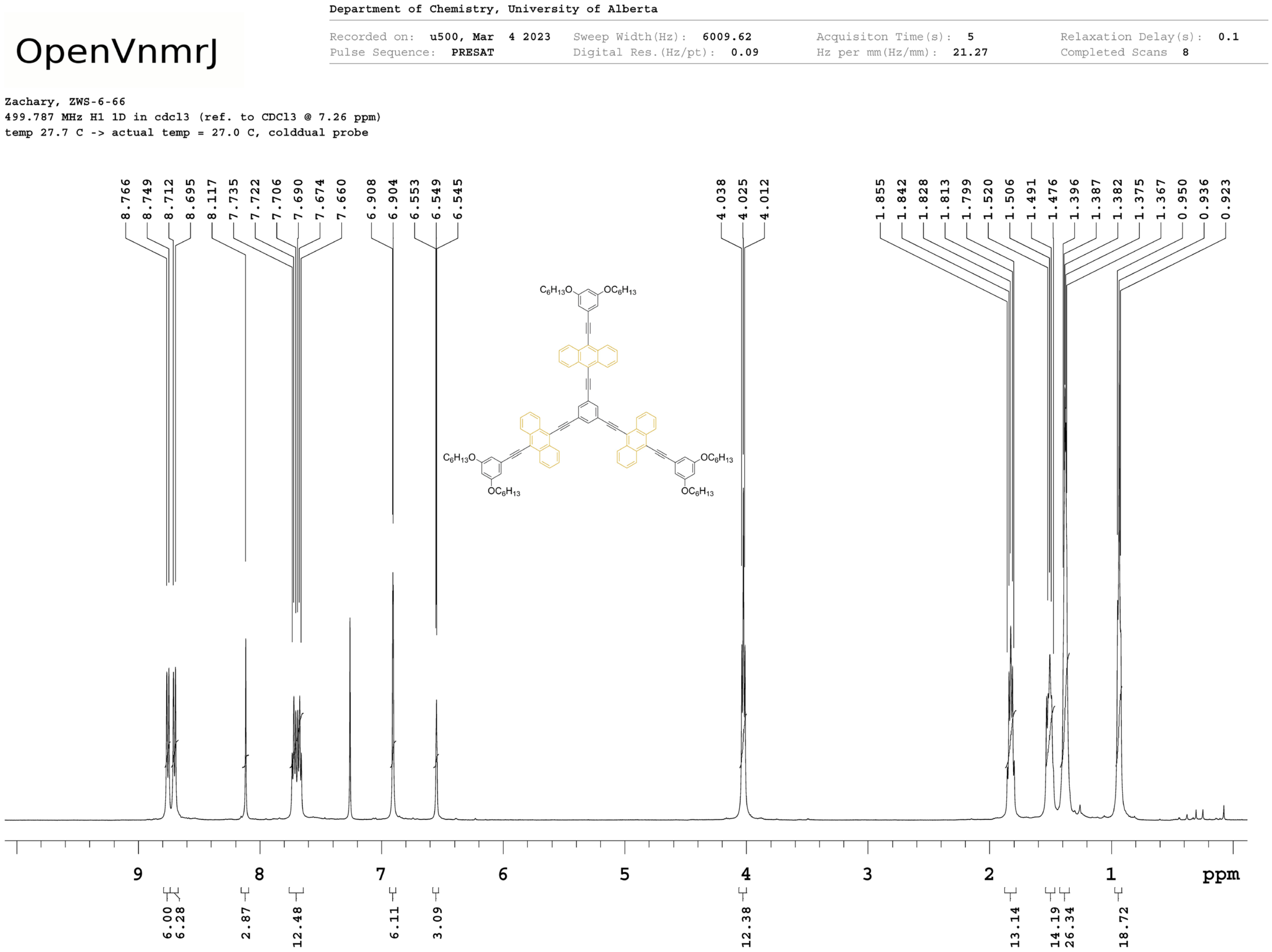

*Figure S38. ^{1}H NMR spectrum (500 MHz) of **BPEAtri** recorded in $CDCl_3$.*

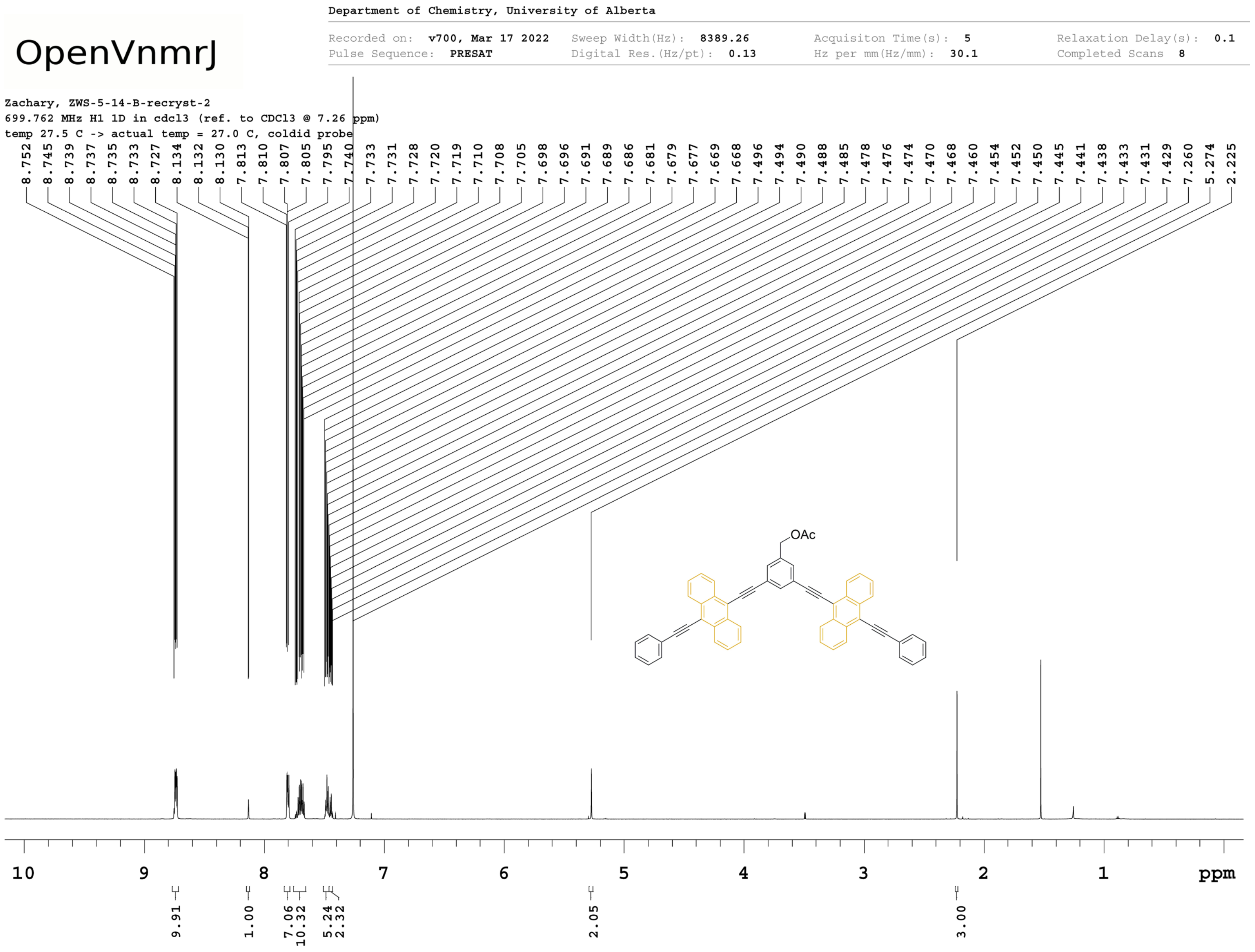

Figure S39. *^{1}H NMR spectrum (700 MHz) of* ***BPEA-Ac*** *recorded in $CDCl_3$.*

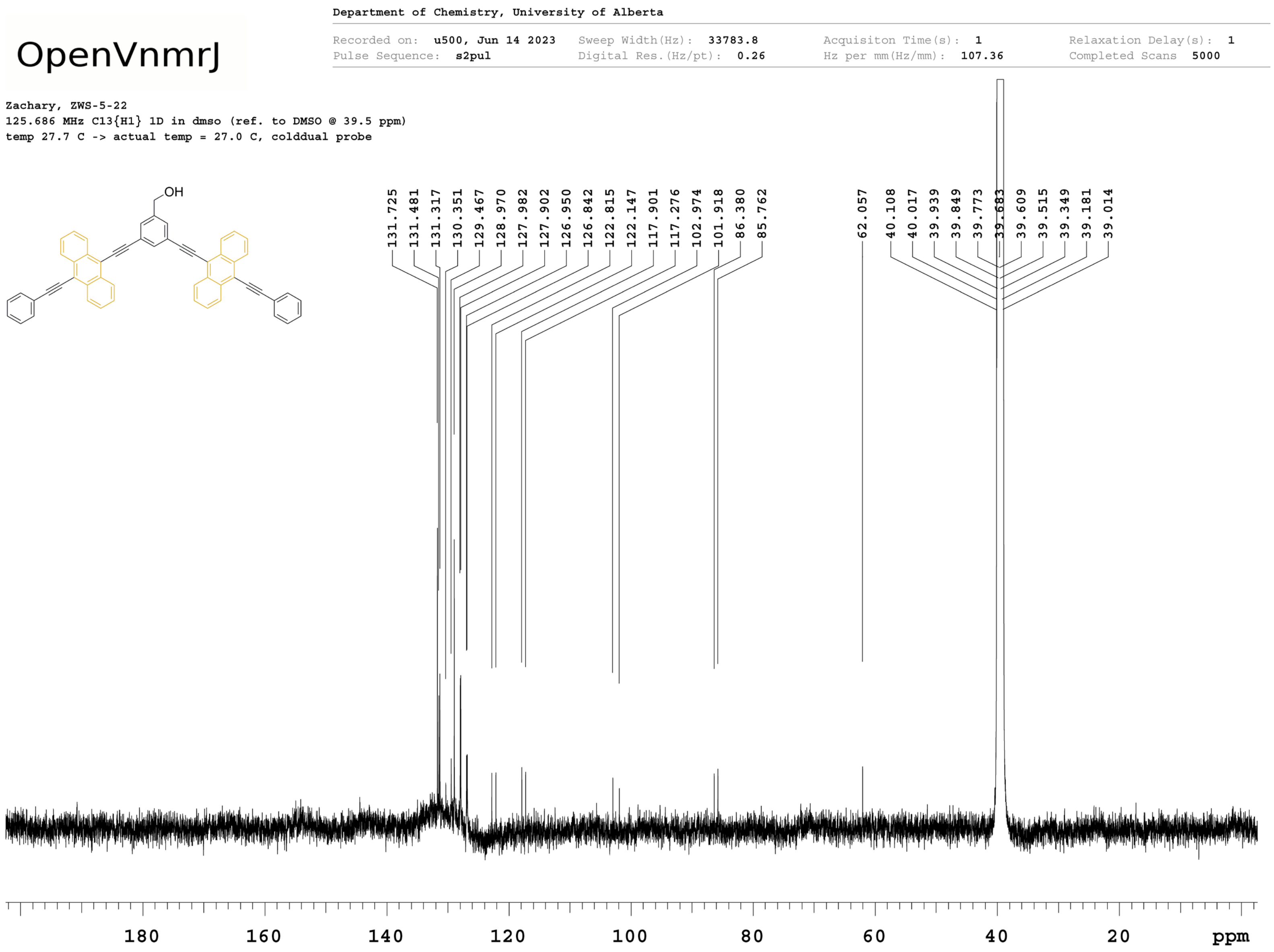

Figure S40. $^{13}C\{^1H\}$ *NMR spectrum (126 MHz) of* ***BPEAdim*** *recorded in DMSO-*d_6.

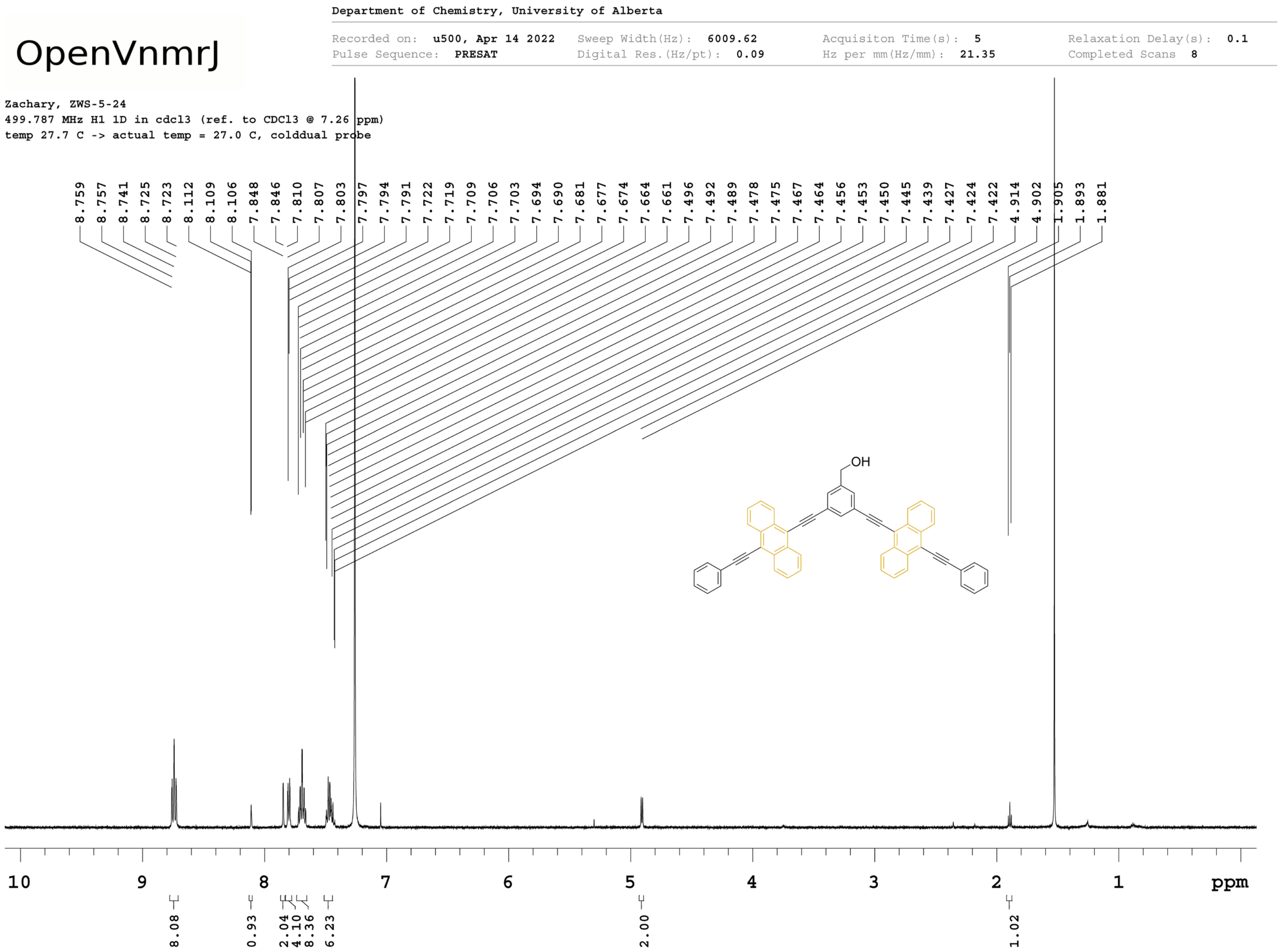

Figure S41. [1]*H NMR spectrum (500 MHz) of* ***BPEAdim*** *recorded in* $CDCl_3$.

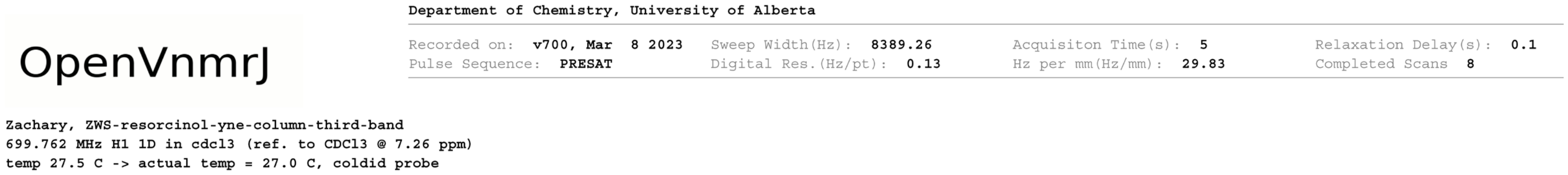

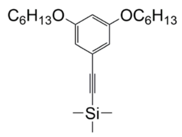

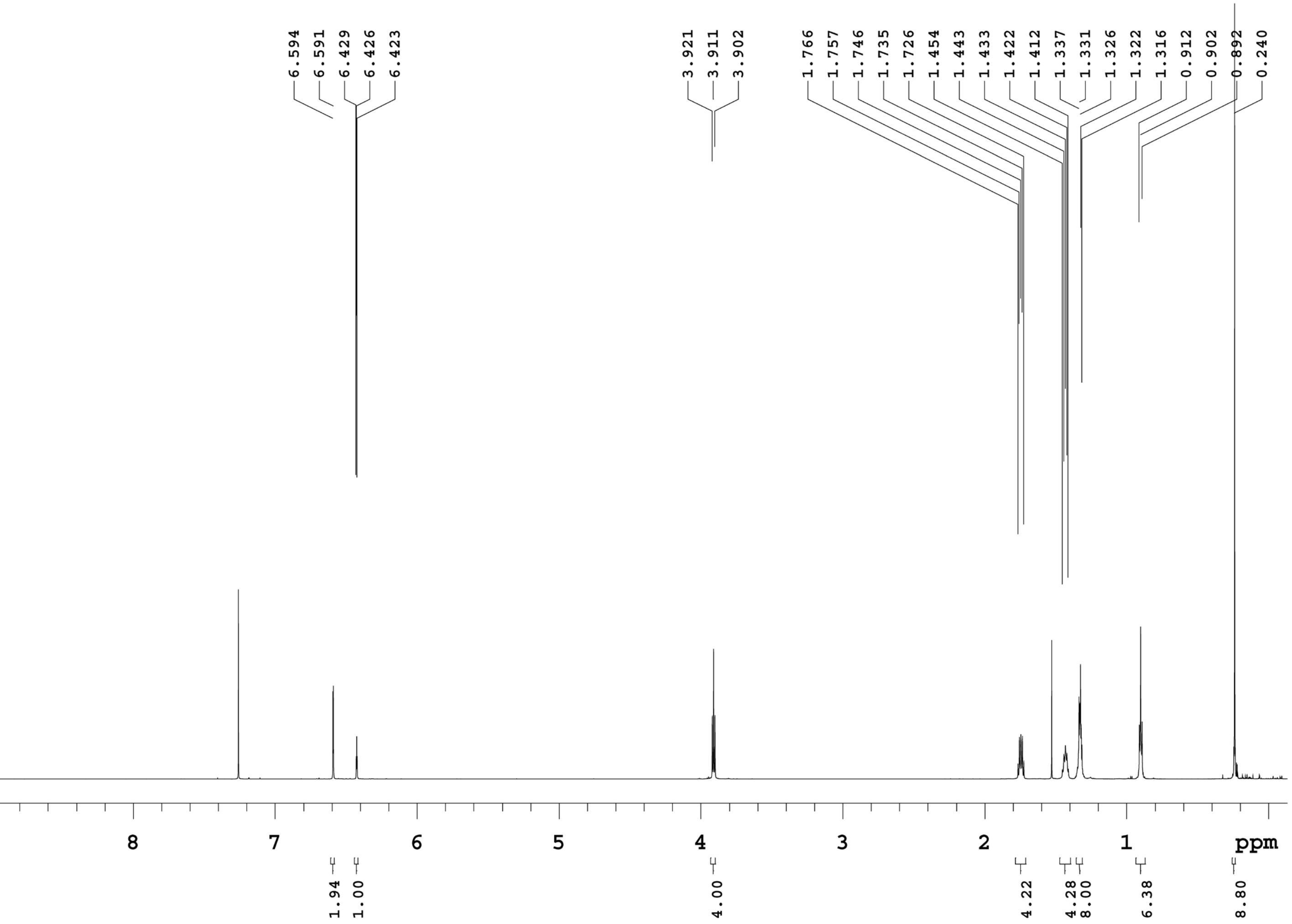

Figure S42. ^{1}H *NMR spectrum (700 MHz) of* ***S10*** *recorded in* $CDCl_3$.

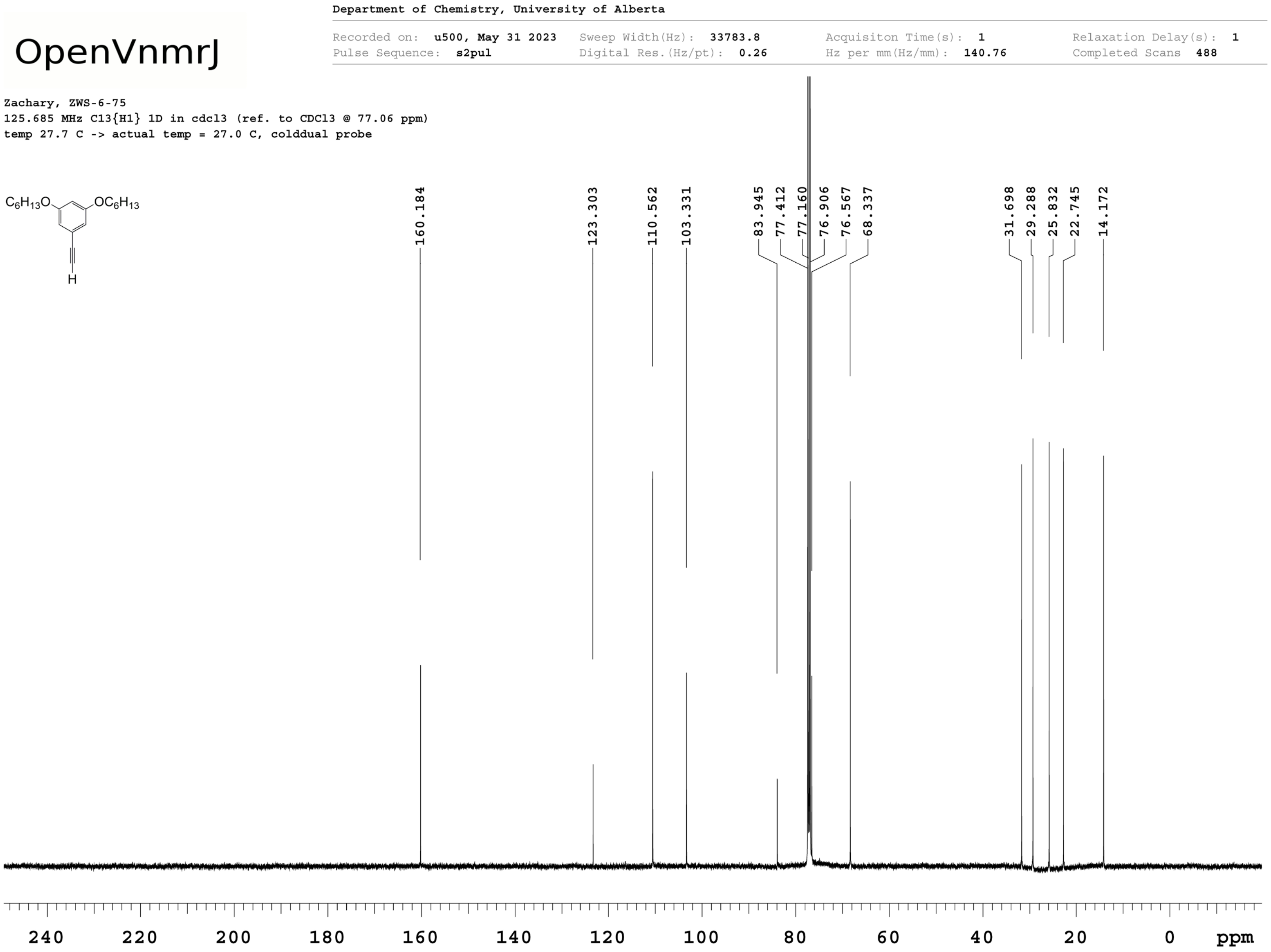

Figure S43. $^{13}C\{^{1}H\}$ *NMR spectrum (126 MHz) of* ***S11*** *recorded in* $CDCl_3$*.*

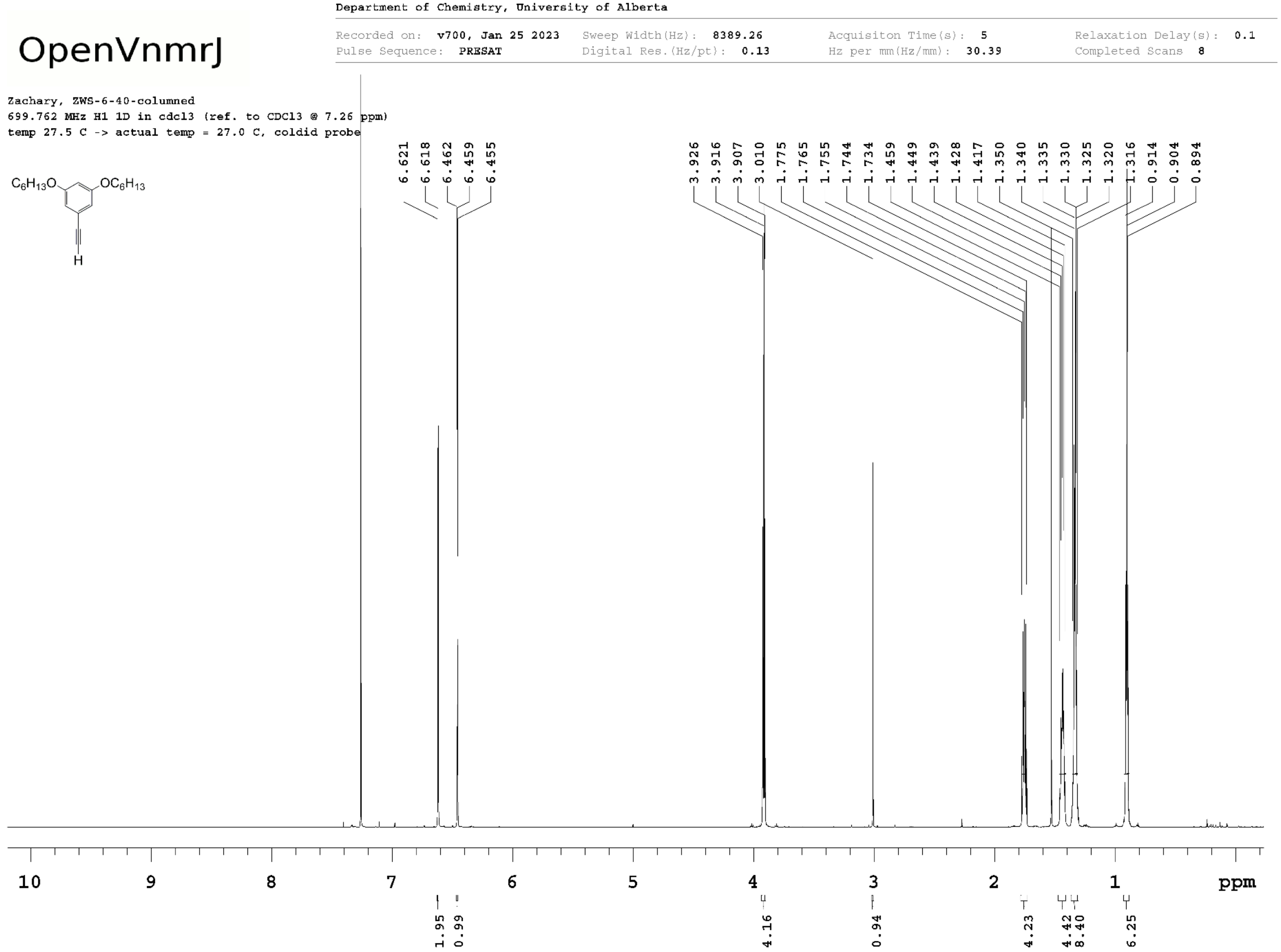

*Figure S44. ^{1}H NMR spectrum (700 MHz) of **S11** recorded in $CDCl_3$.*

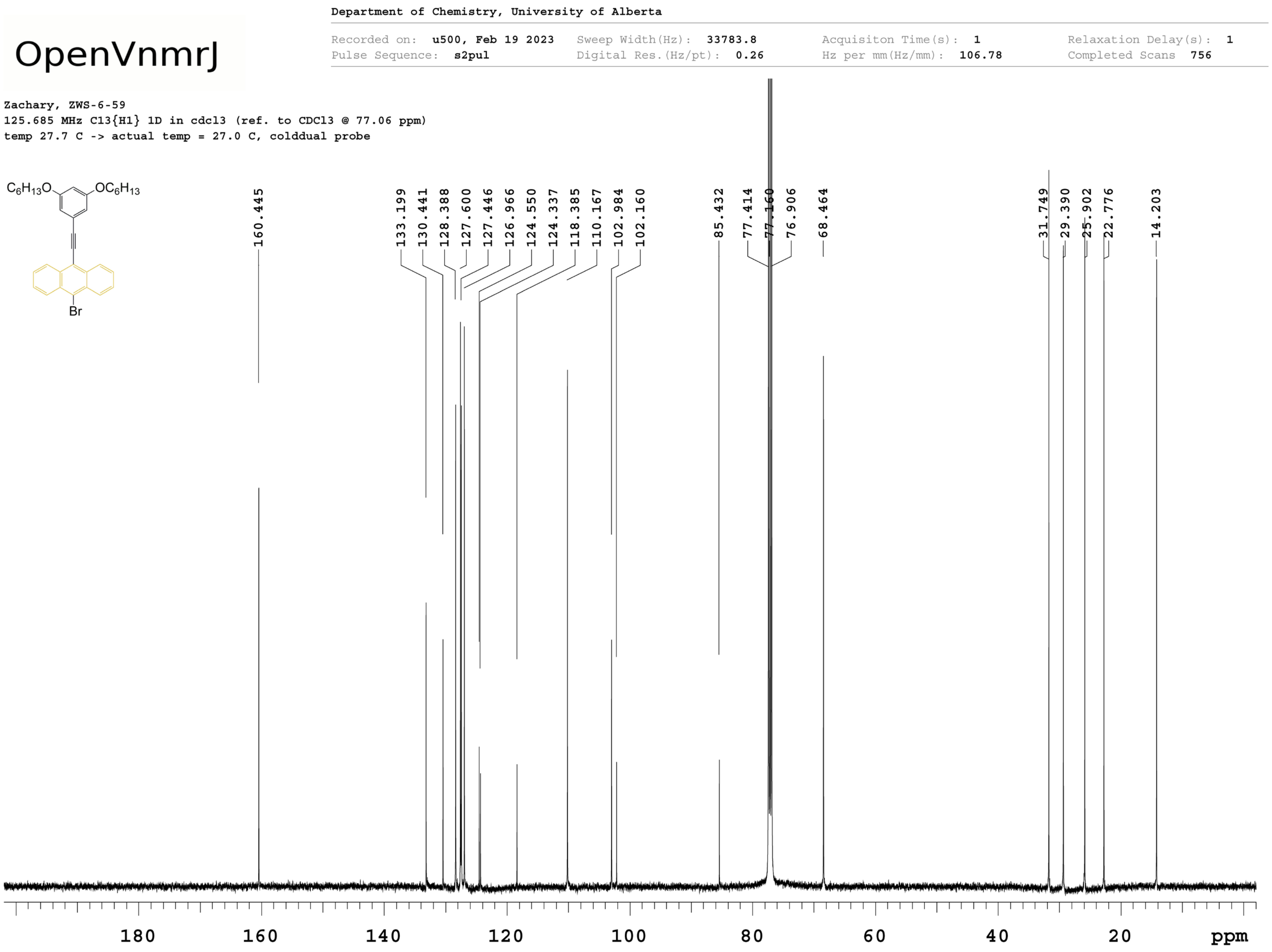

*Figure S45. $^{13}C\{^{1}H\}$ NMR spectrum (126 MHz) of **S3** recorded in $CDCl_3$.*

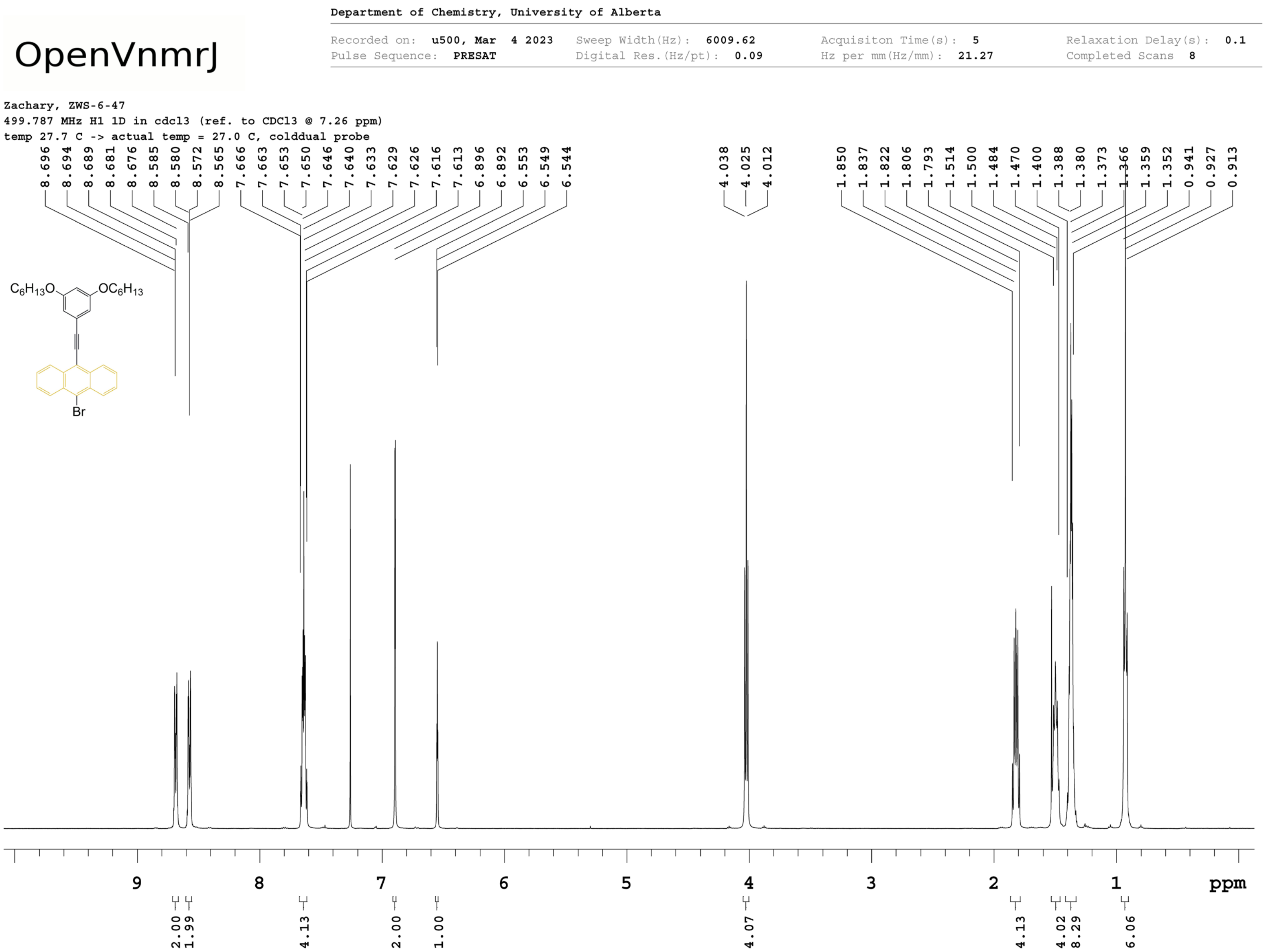

Figure S46. *^{1}H NMR spectrum (500 MHz) of **S3** recorded in $CDCl_3$.*

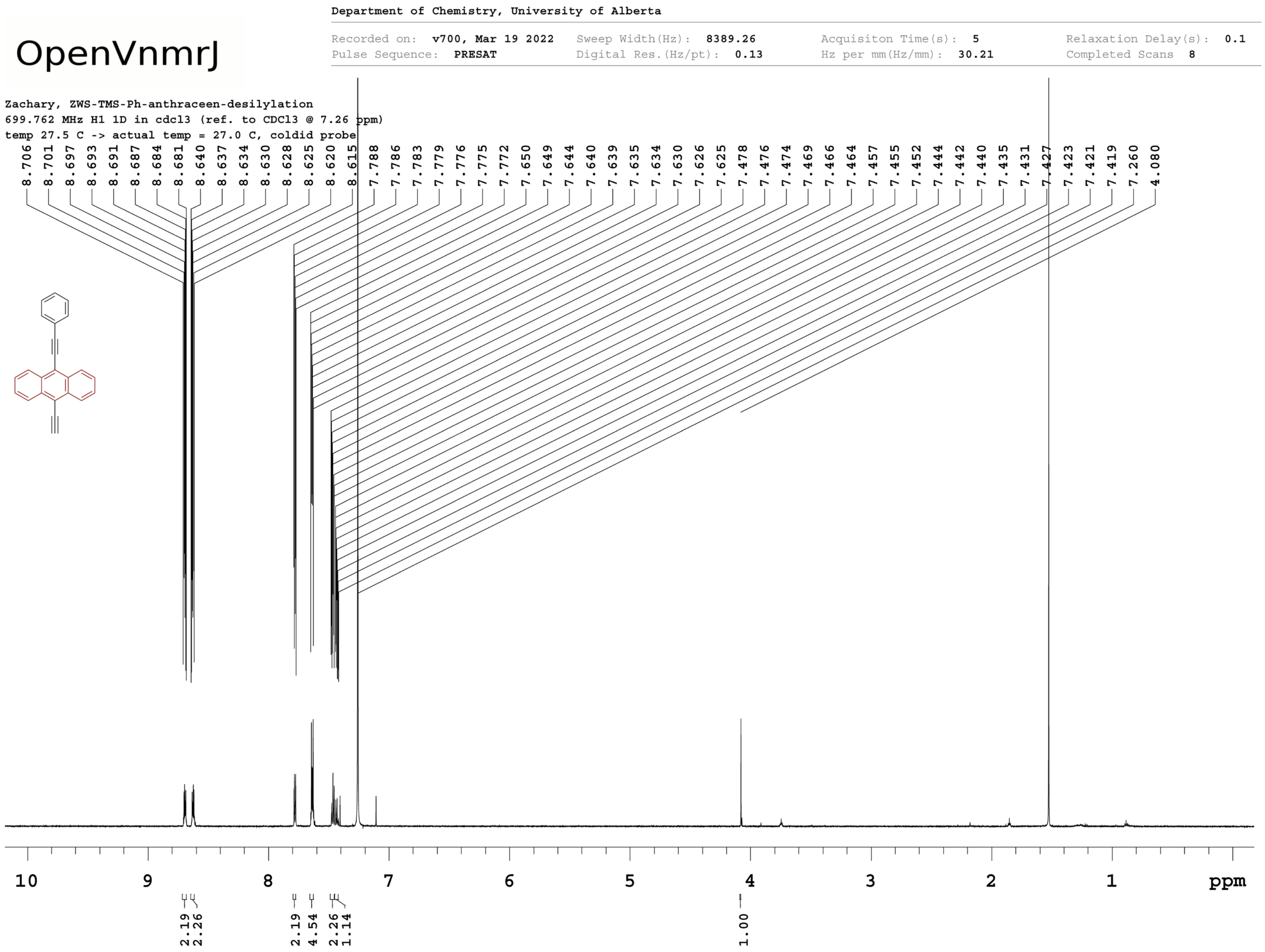

*Figure S47. ^{1}H NMR spectrum (700 MHz) of **S5** recorded in $CDCl_3$.*

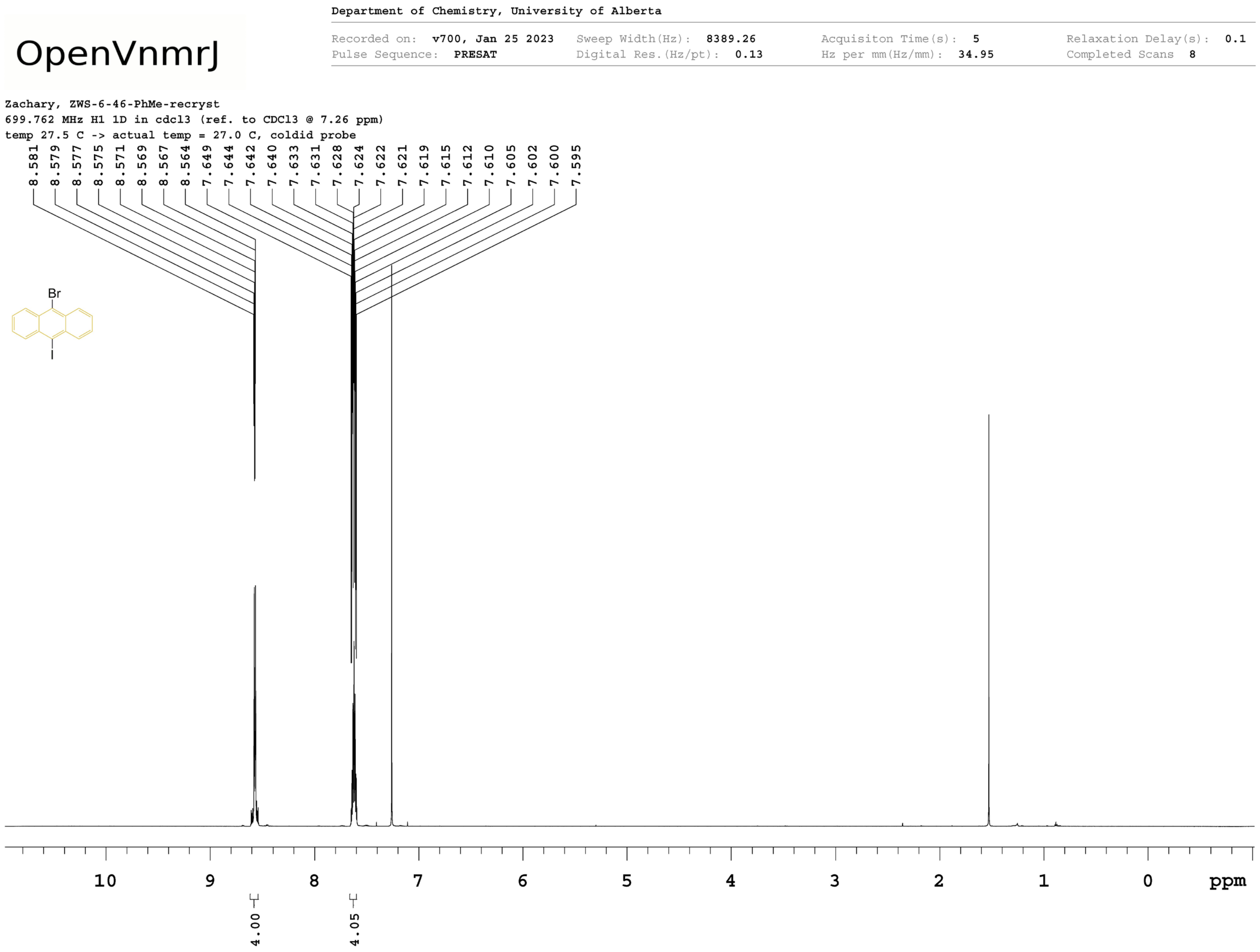

*Figure S48. ^{1}H NMR spectrum (700 MHz) of **S12** recorded in $CDCl_3$.*

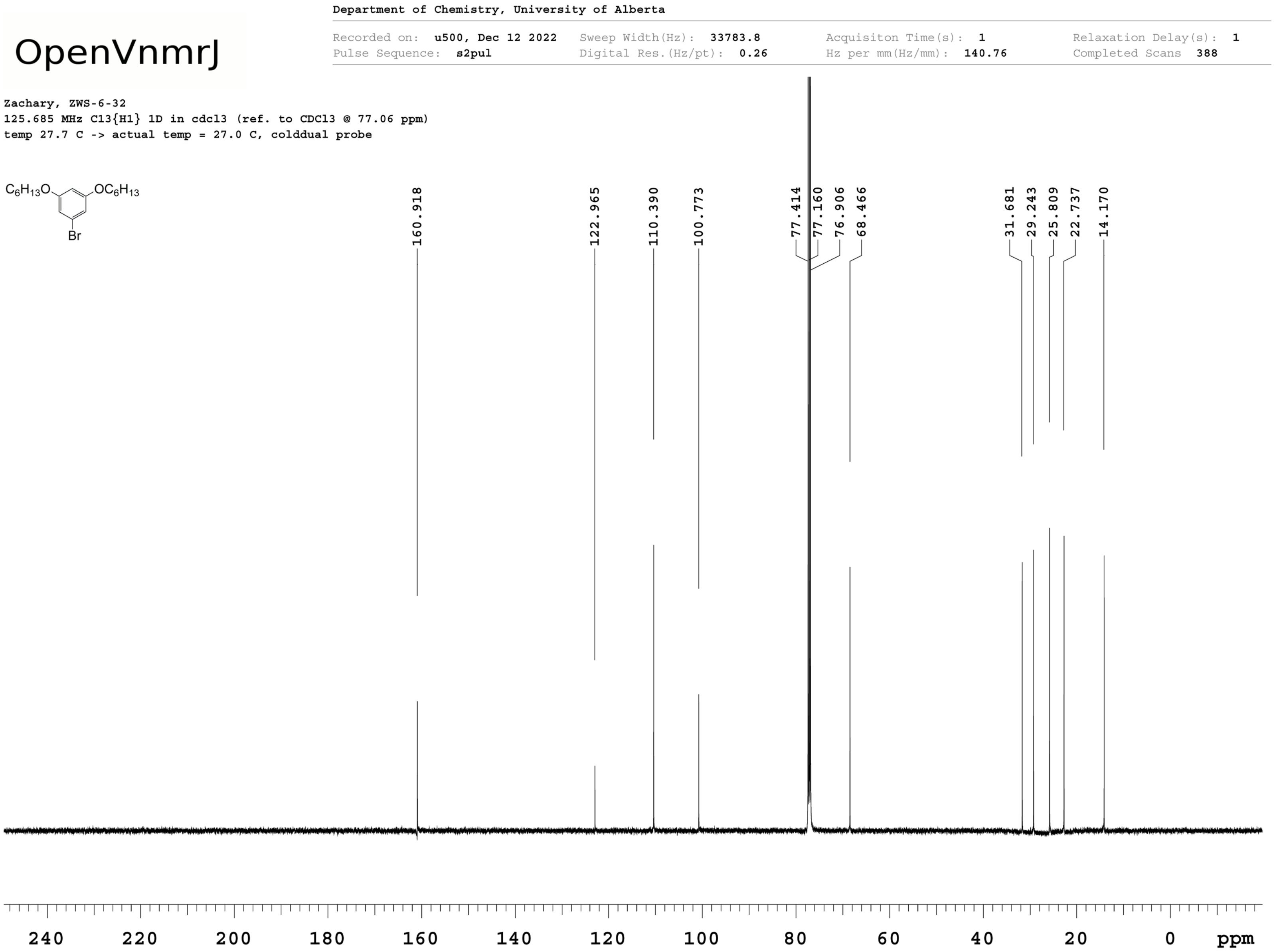

*Figure S49. $^{13}C\{^{1}H\}$ NMR spectrum (126 MHz) of **S9** recorded in $CDCl_3$.*

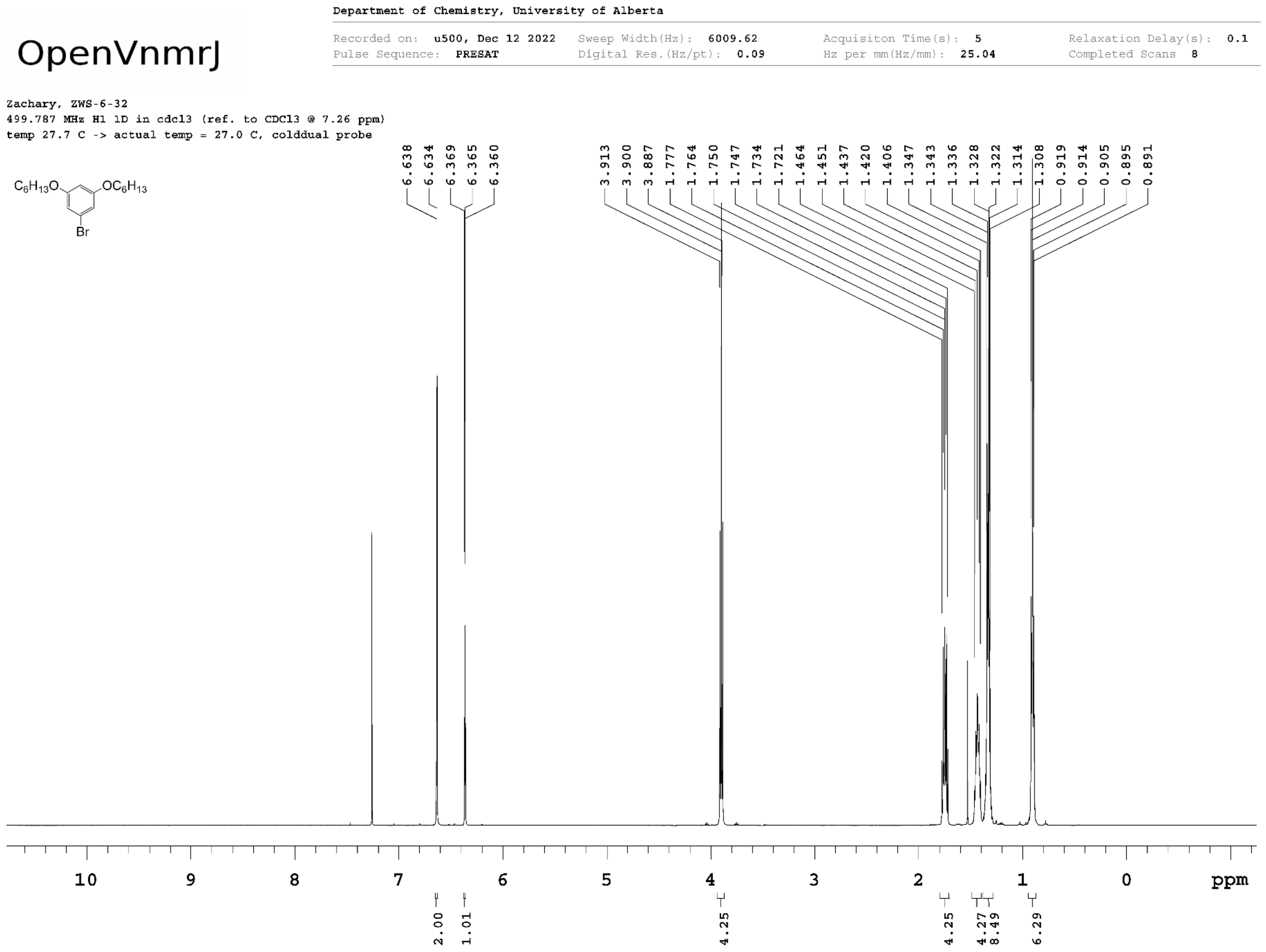

*Figure S50. ^{1}H NMR spectrum (500 MHz) of **S9** recorded in $CDCl_3$.*

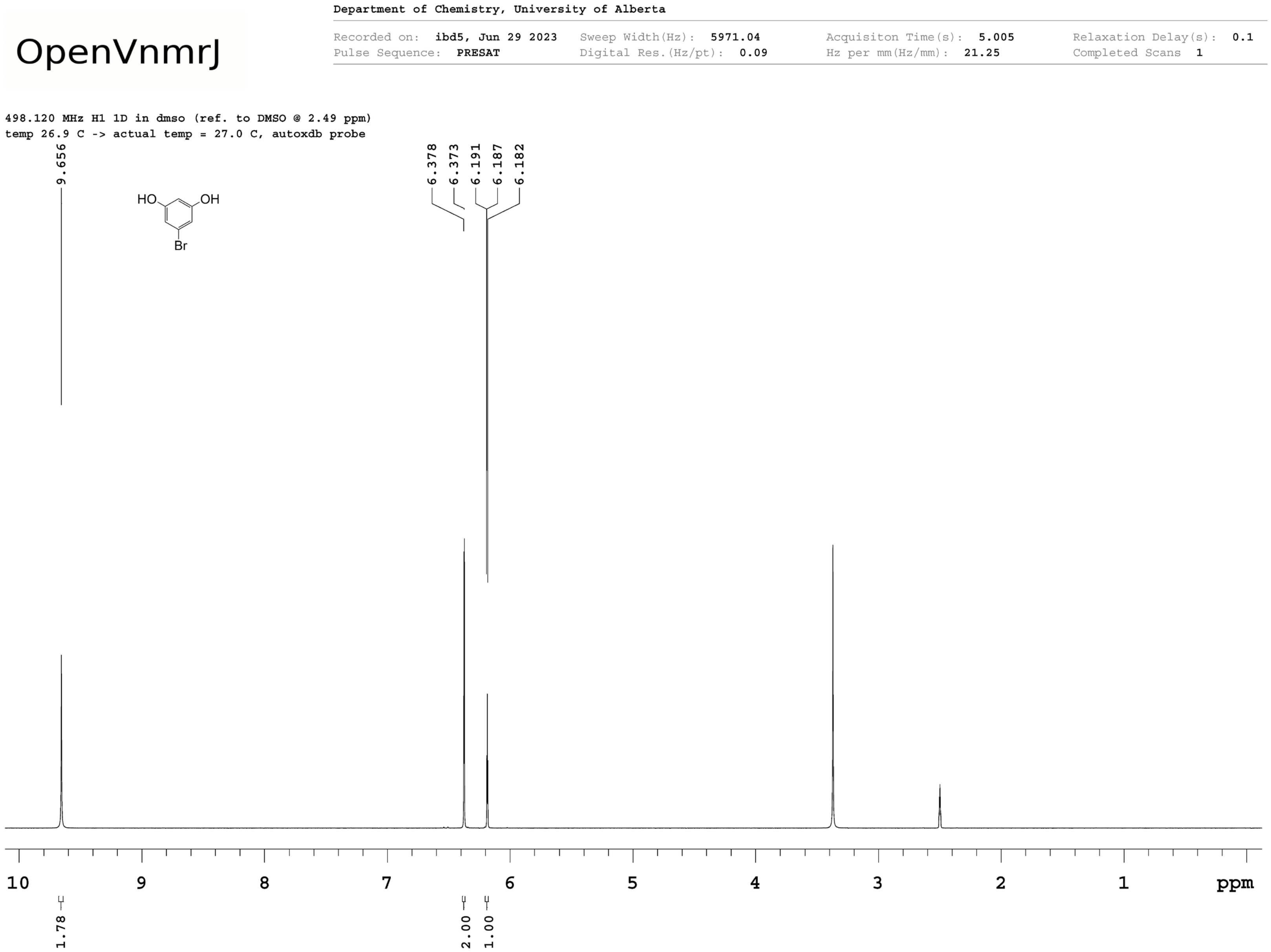

*Figure S51. ^{1}H NMR spectrum (500 MHz) of **S8** recorded in DMSO-d$_6$.*

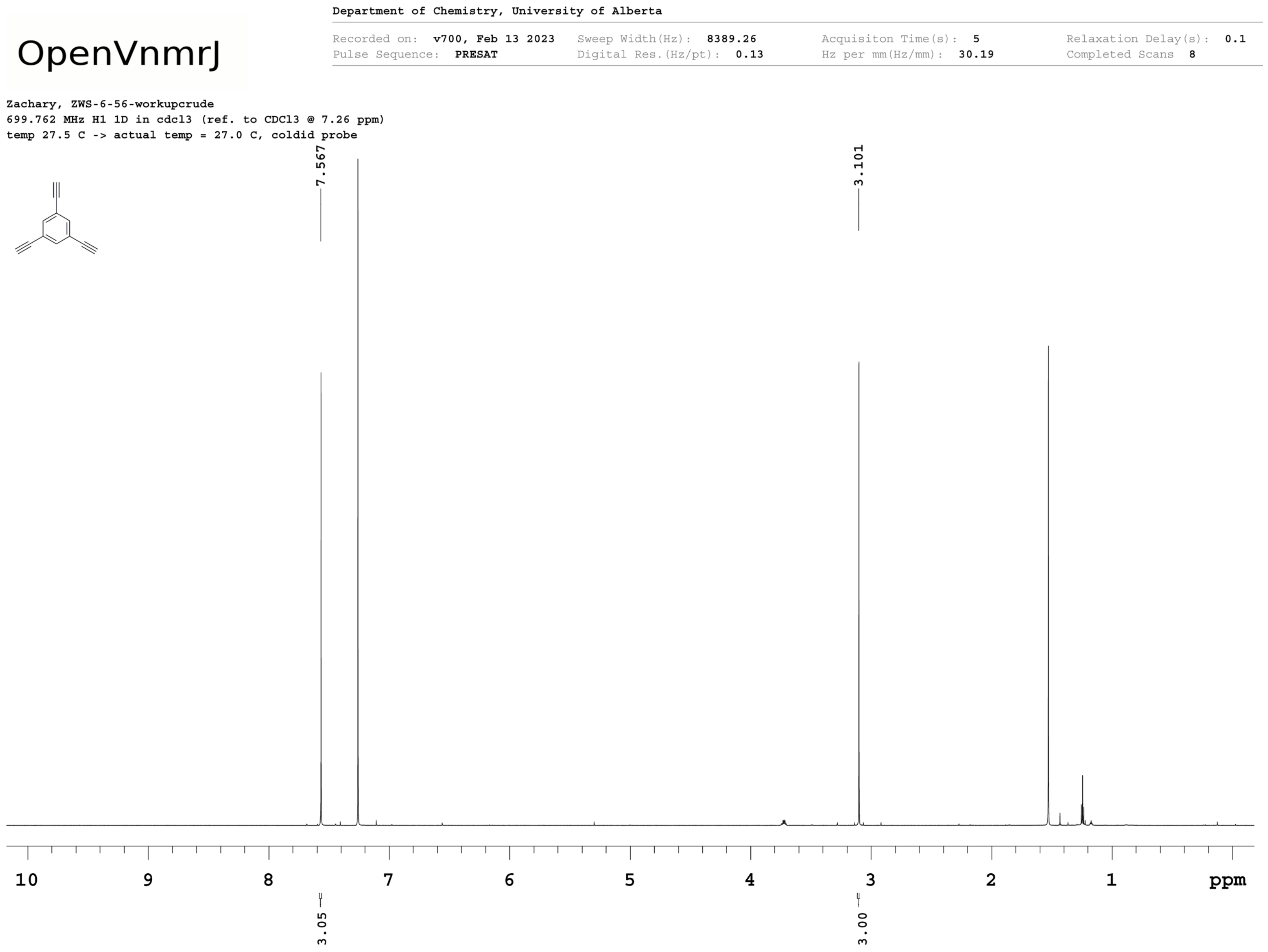

*Figure S52. ^{1}H NMR spectrum (700 MHz) of **S4** recorded in $CDCl_3$.*

9 References

[1] Oheim, M.; Salomon, A.; Weissman, A.; Brunstein, M.; Becherer, U., Calibrating Evanescent-Wave Penetration Depths for Biological TIRF Microscopy. *Biophysical Journal* **2019,** *117* (5), 795-809.

[2] Xue, T.; Zhao, D.; Hao, T.; Li, X.; Wang, T.; Nie, J., Synthesis, one/two-photon optical and electrochemical properties and the photopolymerization-sensitizing effect of anthracene-based dyes: influence of the donor groups. *New Journal of Chemistry* **2019,** *43* (17), 6737-6745.

[3] Weller, A., Photoinduced Electron Transfer in Solution: Exciplex and Radical Ion Pair Formation Free Enthalpies and their Solvent Dependence. *Zeitschrift für Physikalische Chemie* **1982,** *133* (1), 93-98.

[4] Kilså, K.; Macpherson, A. N.; Gillbro, T.; Mårtensson, J.; Albinsson, B., Control of electron transfer in supramolecular systems. *Spectrochim Acta A Mol Biomol Spectrosc* **2001,** *57* (11), 2213-27.

[5] Nesterov, E. E.; Zhu, Z.; Swager, T. M. Conjugation Enhancement of Intramolecular Exciton Migration in Poly(p-Phenylene Ethynylene)s. *J. Am. Chem. Soc.* **2005**, *127* (28), 10083–10088.

[6] Seri, M.; Marrocchi, A.; Bagnis, D.; Ponce, R.; Taticchi, A.; Marks, T. J.; Facchetti, A. Molecular-Shape-Controlled Photovoltaic Performance Probed via Soluble π-Conjugated Arylacetylenic Semiconductors. *Adv. Mater.* **2011**, *23* (33), 3827–3831.

[7] Su, P.; Liu, H.; Shen, L.; Zhou, J.; Wang, W.; Liu, S.; Nie, X.; Li, Z.; Liu, Z.; Chen, Y.; Li, X. Linker Dependent Symmetry Breaking Charge Separation in 9,10-Bis(Phenylethynyl)Anthracene Dimers. Mater. Chem. Front. **2022**, 6 (6), 707–717.

[8] Ma, C.-Q.; Pisula, W.; Weber, C.; Feng, X.-L.; Müllen, K.; Bäuerle, P. Dendritic Oligothiophenes Terminated with Tris(Alkyloxy)Phenylethynyl Tails: Synthesis, Physical Properties, and Self-Assembly. *Chem. - A Eur. J.* **2011**, *17* (5), 1507–1518.

[9] Wen, S.; Yun, X.; Chen, W.; Liu, Q.; Zhu, D.; Gu, C.; Sun, M.; Yang, R. A Triple Bond Side-Chained 2D-Conjugated Benzodithiophene Based Photovoltaic Polymer. *RSC Adv.* **2014**, *4* (102), 58426–58431.

[10] Grunwald, M. A.; Wöhrle, T.; Forschner, R.; Baro, A.; Laschat, S. Columnar Propeller-Like 1,3,5-Triphenylbenzenes: Probing the Effect of Chlorine on the Suzuki Cross-Coupling and Liquid Crystalline Properties. *European J. Org. Chem.* **2020**, *2020* (15), 2190–2198.

[11] Tatum, L. A.; Johnson, C. J.; Fernando, A. A. P.; Ruch, B. C.; Barakoti, K. K.; Alpuche-Aviles, M. A.; King, B. T. Boronic Esters: A Simple Route to Discotic Liquid Crystals That Are Electron Deficient. *Chem. Sci.* **2012**, *3* (11), 3261–3264.

[12] Mangione, M. I.; Spanevello, R. A.; Rumbero, A.; Heredia, D.; Marzari, G.; Fernandez, L.; Otero, L.; Fungo, F. Electrogenerated Conductive Polymers from Triphenylamine End-Capped Dendrimers. *Macromolecules* **2013**, *46* (12), 4754–4763.
